# Off-label and repurposed use of disulfiram beyond alcohol dependence: A systematic review of clinical and preclinical evidence

**DOI:** 10.1016/j.isci.2026.115441

**Published:** 2026-04-03

**Authors:** Beáta-Mária Benkő, Neuza Sofia De Brito Parreirinha, István Sebe, Romána Zelkó

**Affiliations:** 1 University Pharmacy Department of Pharmacy Administration, Semmelweis University, Hőgyes Endre u. 7-9., 1092 Budapest, Hungary; 2 Center for Pharmacology and Drug Research & Development, Semmelweis University, Budapest, Hungary; 3 Faculty of Pharmacy, University of Lisbon, Av. Prof. Gama Pinto 1649-003 Lisboa, Portugal

**Keywords:** Drugs, Safety assessment, Cancer

## Abstract

This systematic review assesses off-label and repurposed uses of disulfiram (DIS) beyond alcohol dependence, synthesizing clinical trials, case reports, and preclinical studies (2015–2025) per PRISMA 2020 guidelines. Database and registry searches yielded 229 studies: 44 clinical trials (mainly cancer, infectious diseases) and 185 articles. Cancer-led indications, followed by infectious, inflammatory, cognitive, and metabolic diseases. Off-label use is under-documented; trials often failed due to recruitment issues, poor responses, adverse events, or funding. They yielded data on high-dose regimens and safety, stressing risk-benefit assessments. Preclinical promise faces clinical hurdles such as poor bioavailability, formulation limits, and high-dose toxicity. Key barriers: (1) low oral bioavailability and rapid hepatic metabolism, (2) unscaled delivery systems, (3) adverse events needing evaluation. DIS holds life cycle potential via multifaceted mechanisms; success demands optimized delivery, rigorous trials, and scalable formulations.

## Highlights

• Preclinical-to-clinical matrix of disulfiram repurposing beyond alcohol dependence
• Cancer leads to repositioning, spans infections, inflammation, metabolism, cognition
• Off-label and clinical use reveal dosing, safety, and toxicity concerns
• Unbalanced preclinical mechanistic data and scalable drug development

## Graphical abstract

**Figure:**
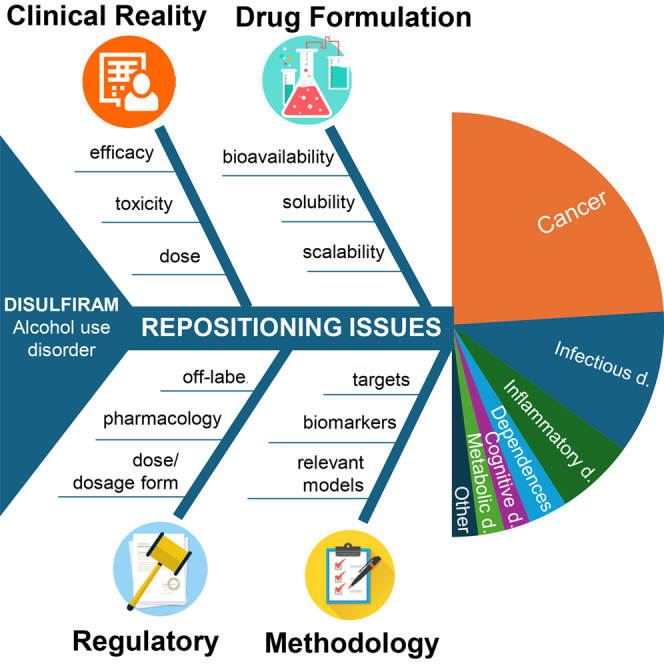

## Introduction

Medicines receive marketing authorization for specific indications and dosages following rigorous testing, most commonly through randomized controlled trials. If the trials succeed, a license is granted, and the medication is marketed with a summary of product characteristics (SmPC), referred to as “label.”^1^ A drug’s life cycle continues to evolve after marketing approval through pharmacovigilance strategies. These strategies focus on detecting, assessing, understanding, and preventing adverse effects or other drug-related problems, as mandated by pharmaceutical legislation.^2^^,^^3^^,^^4^ Pharmaceutical companies can expand their products' usage potential through further investment, for example, by extending therapeutic indications, but this requires a new authorization process. Although new patents and regulatory protection can be obtained for an extension of indication, current clinical practice shows frequent prescribing of medicines for the extended indications, even though the products are not authorized for these new therapeutic indications.^5^

The off-label use, not covered by the pharmaceutical legislation, but recognized as a concept by law (i.e., pharmacovigilance provisions in Directive 2010/84/EU), is considered as an approach of drug life cycle extension. This term is used for the prescription of a medicinal product for any indication, patient group, route of administration, dosage, or treatment regimen other than that listed in the SmPC.^1^ The marketing authorization holders are required to collect and report information on suspected adverse reactions arising from the use of medicines outside the terms of the marketing authorization; however, when doctors prescribe unapproved medicines, they bear full responsibility for any adverse patient outcomes, even with signed informed consent.^4^

Semantically, a related term to off-label use is drug repositioning (also referred to as drug repurposing, reprofiling, redirecting, switching, and so forth).^6^ Although some historical examples exist, this is a relatively recent concept, defined in 2004 by Ashburn and Thor as a process of finding new uses outside the scope of the original medical indication for existing drugs.^6^^,^^7^ The original drug repositioning definition has expanded to encompass active substances that failed clinical development due to toxicity or poor efficacy, as well as drugs withdrawn from the market over safety issues.^6^ In contrast with off-label use, drug repositioning undergoes an authorization process, but with shortcuts.^8^ While traditional or *de novo* drug discovery is a time-consuming, costly, and high-investment process, the authorization procedure of an old repurposed drug takes into account data previously acquired, e.g., safety and toxicity data, which can make the initial phases considerably faster, and therefore cheaper, and increases the chances of introducing it on the market.^6^^,^^8^ However, any change in formulation, dosage, or administration route demands the reassessment of its safety profile under these modified conditions, constituting a new medicinal product.^6^

Disulfiram (DIS), with its particular drug discovery history, is a representative candidate to evaluate how off-label use and drug-repositioning strategies influence the life cycle of an old drug. In the 20th century, DIS was introduced in the rubber industry to accelerate sulfur vulcanization, and due to a series of chance discoveries, from the early 1940s it was also used as a scabicide, and in 1951 it was approved for chronic alcoholism treatment, where it has remained in use for more than 70 years.^9^ DIS acts by irreversibly inhibiting the enzyme aldehyde dehydrogenase (ALDH), which converts acetaldehyde (a toxic ethanol metabolite) into acetate. When this conversion is blocked, acetaldehyde accumulates after alcohol intake, triggering unpleasant symptoms such as nausea, flushing, tachycardia, and general discomfort, thereby discouraging alcohol consumption.^10^ Since naltrexone and acamprosate emerged as alternatives, DIS has declined in popularity for treating alcohol use disorder.^9^ Yet DIS retains relevance across clinical interest, with ongoing discoveries of new mechanisms and applications, in clinical areas such as oncology, infectious diseases, inflammatory diseases, neurology, and addiction beyond alcohol use therapy.^9^

The scientific, regulatory, and ethical considerations highlight the importance of systematic reviews that critically assess the quality and therapeutic relevance of available evidence on DIS use beyond its approved indication. DIS presents a paradox: more than 70 years of clinical use and extensive preclinical support across multiple diseases, yet no new therapeutic indications have been approved despite major research investment over the past decade. While many preclinical studies support DIS’s potential in diverse disease models, the extent to which this translates into real-world clinical use remains unclear. A critical gap exists between *in vitro* efficacy and *in vivo* clinical outcomes, primarily attributable to pharmacokinetic limitations (suboptimal bioavailability, rapid metabolism) and formulation challenges rather than a lack of mechanistic understanding. Therefore, this systematic review aims to distinguish between theoretical repositioning and actual clinical application, to identify and critically analyze the scientific evidence supporting the reposition and off-label use of DIS in therapeutic contexts other than alcohol dependence. This review seeks to clarify the current landscape of the DIS life cycle, evaluating the intersection between off-label use and drug repurposing.

## Results

### Trends of disulfiram use beyond alcohol dependence

**Figure 1:**
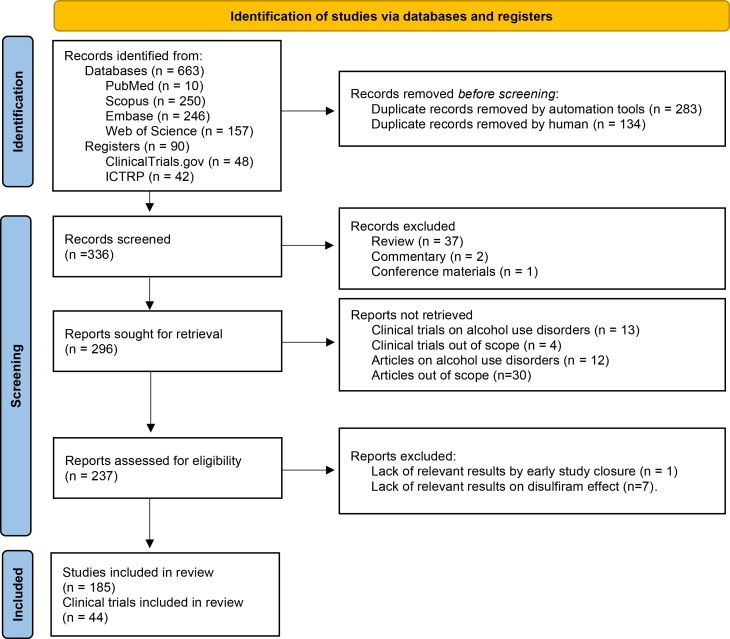
PRISMA flow diagram for systematic reviews PRISMA: Preferred Reporting Items for Systematic Review and Meta-analysis.

**Figure 2:**
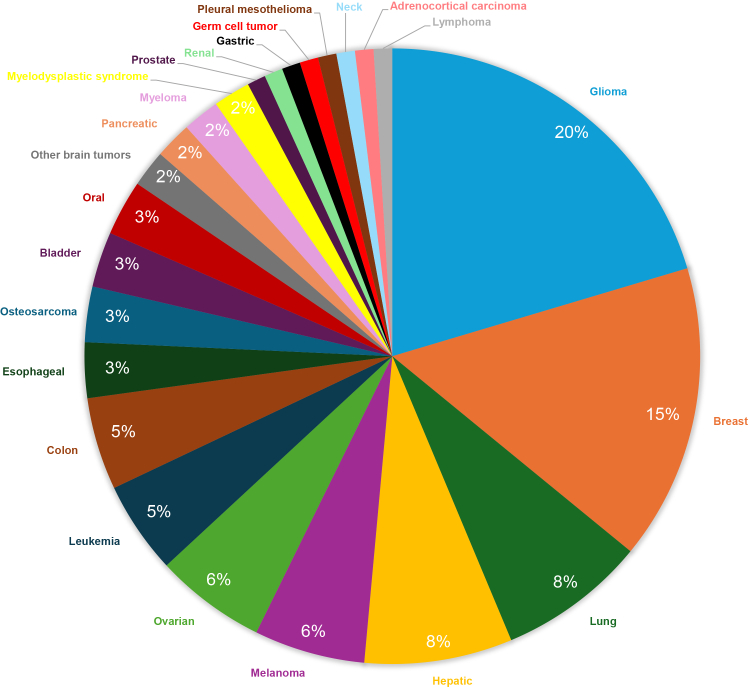
Frequency of tumor type model utilization in original research articles (2015–2025) investigating disulfiram repurposing for anticancer therapy |This pie chart shows the proportion of original research articles published between 2015 and 2025 that investigated disulfiram repurposing for anticancer therapy, grouped by the tumor type model used (e.g., glioma, breast cancer, and lung cancer). Each slice represents the frequency share of articles for a given tumor type, highlighting which cancer models have been most frequently studied in the context of disulfiram-based anticancer strategies.

**Figure 3:**
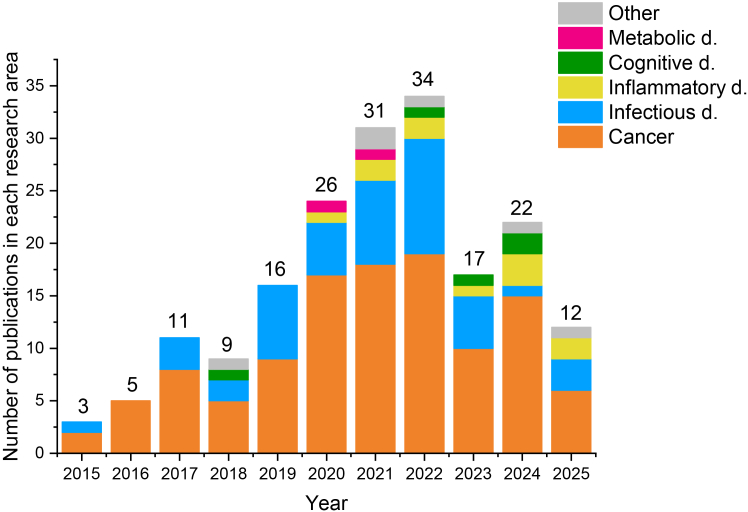
Annual publication trends of studies on disulfiram beyond alcohol dependence (January 1, 2015 – December 15, 2025) This bar chart shows the annual number of original research articles published between January 1, 2015, and December 15, 2025, that investigated disulfiram in indications other than alcohol dependence. Each bar is subdivided by disease category (e.g., cancer, infectious diseases, metabolic disorders, and others), illustrating both the overall publication trend over time and the changing ratio of disease-type focus across years. Abbreviations: d = diseases.

**Figure 4:**
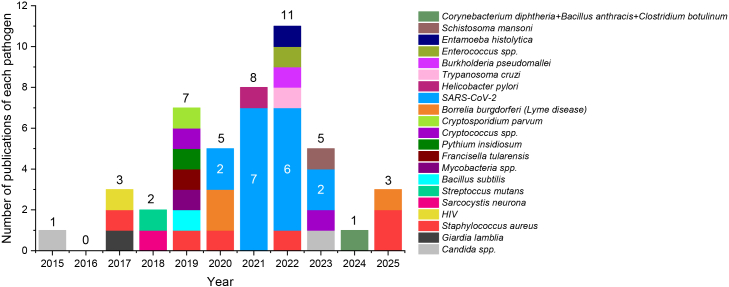
Annual number of publications on infectious diseases, stratified by individual pathogens (January 1, 2015 – December 15, 2025) This bar chart displays the yearly number of original research articles published between January 1, 2015, and December 15, 2025, that investigated infectious diseases, grouped by individual pathogen (e.g., Candida spp., Borrelia burgdorferi, SARS-CoV-2, Giardia lamblia, and others). Each bar represents the total publication count for a given year, and the stacked segments correspond to different pathogens, illustrating how research focus shifts across pathogens over time.

This systematic review was conducted with the objective of synthesizing and critically evaluating the scientific evidence supporting the repositioning and off-label use of DIS in therapeutic contexts beyond alcohol dependence. Although interest in DIS has increased over the past decade - driven largely by preclinical findings suggesting antitumor, antimicrobial, and anti-inflammatory properties - no new regulatory approvals have yet been granted for alternative indications. The literature search revealed 753 records from 6 databases, 417 were excluded because of duplication, and 336 were screened according to the eligibility criteria. A total of 98 hits were declined as they were either reviews, expert opinions, commentary, conference materials, or not suitable for the topic under review. A total of 237 records were reviewed fully, and only 229 were deemed suitable for inclusion (Figure 1). The 44 clinical trials are summarized in Table 1 and the 185 research articles in Table S1. The number of original articles published shows a growing tendency of interest in DIS repositioning, mainly in oncology. The antitumor effect of DIS was discovered as early as 1977, and since then, approximately half a decade of research has been conducted, but there has still been no clinical breakthrough.^11^ The most explored tumor types are glioma and breast cancer, obviously due to the severity and hard-to-treat nature of brain tumors, and due to the first case report and clinical trial investigating DIS for cancer, both targeting the tumors of the breast^9^^,^^11^ (Figure 2). Although infectious diseases are only the second most frequently researched alternative treatment scope of DIS, this is not a new tendency of therapeutic applications, as its antiparasitic effect was studied before its anti-alcohol effect was discovered.^12^ The method of identifying new therapeutic uses for existing and approved compounds has gained renewed attention in recent years, especially during the coronavirus disease 2019 (COVID19) pandemic, when several approved drugs (e.g., remdesivir) were rapidly studied for new indications.^13^^,^^14^ These developments have reinforced the value of drug repurposing in accelerating access to therapies with known safety profiles.^13^ The impact of COVID19 on drug repositioning is also recognizable in publications trends of DIS^15^: starting in 2019, the number of publications doubled by 2022, after which the annual number of publications declined again (Figure 3). In this time slot, the number of publications on infectious diseases increased, including those exploring DIS as a potential COVID-19 therapy (Figure 4). Although the overwhelmed drug repositioning strategies had a debatable impact on DIS research. The drug repurposing methods acquired a negative connotation due to the promotion of ineffective drugs during COVID19 with flawed data extrapolation from *in vitro* studies, reliance on poorly peer-reviewed or predatory journals, shoddy experiments, and social media conspiracy theories, which fostered “unscientific” views over expert evidence-based advice, amplifying misinformation to a vulnerable public.^15^ Over the past five years, research on DIS has extended to inflammatory diseases, cognitive and metabolic disorders. DIS`s therapeutic potential for diseases other than alcoholism illustrates how older, well-established drugs can be reconsidered considering emerging evidence and evolving public health challenges.^9^ By prioritizing documented administration and measurable outcomes, the review aimed to clarify the extent to which DIS is being effectively used beyond its approved indication. Thus, the following sections separately review the state-of-the-art evidence on the off-label and repurposed uses of the drug.

**Table 1:** Clinical evidence of disulfiram uses beyond alcohol dependence, according to ClinicalTrials.gov and ICTRP databases from 2025 November, searching with the disulfiram intervention query and limiting the study records updates to the last decade

No.	Registration number	Title	Conditions	Disulfiram intervention	Phase	Registration date	Status	Aim and Results (if applicable)
**Cancer**								

1.	NCT01777919	Disulfiram/Copper Combination In The Treatment of Newly Diagnosed Glioblastoma Multiform	glioblastoma	Daily administration of disulfiram and copper will take place for the whole study period	II	2025	Not yet recruiting	The purpose of this study is to test the disulfiram/copper combination as an adjunctive and concurrent chemotherapy in the treatment of newly diagnosed glioblastoma.
2.	NCT05210374	Disulfiram With Copper Gluconate and Liposomal Doxorubicin in Treatment-Refractory Sarcomas	relapsed sarcomas	Disulfiram is taken orally daily in the AM (max 480 mg/day)	I	2023	Recruiting	The purpose of this study is to test the safety of combining the disulfiram and copper gluconate to liposomal doxorubicin to treat patients with sarcomas that recurred or did not respond to initial treatment.
3.	NCT05667415	Treatment of Advanced Gastric Cancer with Disulfiram	gastric cancer	400 mg of disulfiram orally every day	N/A	2022	Not yet recruiting	In this clinical study, cisplatin combined with disulfiram is mainly used to treat advanced gastric cancer.
4.	NCT04521335	Study of Disulfiram and Copper Gluconate in Patients With Treatment-Refractory Multiple Myeloma	multiple myeloma	Twice daily at the assigned dose level	I	2021	Terminated	The trial of disulfiram in combination with copper gluconate in patients with treatment-refractory multiple myeloma opens the evaluation with dose escalation, followed by an expansion cohort to further characterize the safety and tolerance of the combination.
5.	EUCTR2019-001957-16-SK and NCT04265274	Study to evaluate the effectiveness and toxicity of the combination of vinorelbine, cisplatin, disulfiram, and copper for the treatment of breast cancer with present circulating tumor cells, who did not respond to previous therapy.	breast cancer	Effervescent disulfiram tablet, 400 mg	II	2019	Withdrawn	The study aims to determine the efficacy (as measured by objective response rate) of vinorelbine, cisplatin, and disulfiram and copper in patients with refractory metastatic hormone receptor-positive, HER2-negative, breast cancer CTC_EMT positive. Early termination due to insufficient recruitment.
6.	EUCTR2019-002748-25-DK	Repurposing disulfiram as treatment for metastatic colorectal cancer an investigator initiated a clinical phase II trial	metastatic colorectal cancer	Effervescent disulfiram tablet from 200 to 400 mg	II	2019	Not Recruiting	The study investigates the efficacy and safety of the treatment combination of irinotecan, disulfiram, and copper in patients with metastatic colorectal cancer who have developed resistance to irinotecan.
7.	EUCTR2019-000558-68-SK and NCT03950830	Study to evaluate the effectiveness of disulfiram and cisplatin in patients with refractory testicular cancer.	refractory testicular germ cell cancer	Effervescent disulfiram tablet, 400 mg	II	2019	Completed	The study determines the efficacy (as measured by overall response rate) of disulfiram and cisplatin in patients with multiple relapsed/refractory germ cell tumors. Investigators hypothesize that the inactivation of ALDH by disulfiram recovers cisplatin sensitivity in patients with progressing or relapsing germ cell cancer. Results: None of patients achieved objective response to treatment so the study was terminated in first stage.
8.	JPRN-jRCTs031180183	Phase 1 study of Disulfiram and Nivolumab for gastric cancer	gastric cancer	Disulfiram (po): 160 mg three times daily (480 mg/day) or 80 mg three times daily (240 mg/day) for maximum 6 cycles	I	2019	Completed	The study aims to determine the rate of dose-limiting toxicities expression.
9.	NCT03714555	Disulfiram-Copper Gluconate in Met Pancreas Cancer with Rising CA19-9 on Abraxane-Gemzar, FOLFIRINOX, or Gemcitabine	metastatic pancreatic cancer	Orally, at the assigned dose level	II	2019	Terminated	The study evaluates the effect of Disulfiram + Copper Gluconate in patients’ metastatic pancreatic cancer whose CA-19-9 levels rise while receiving nab-paclitaxel (Abraxane) plus gemcitabine (Gemzar) or FOLFIRINOX or single-agent gemcitabine (Gemzar). One patient was treated for 6 weeks and ended trial participation due to progressive disease.
10.	NCT03363659	Disulfiram and Copper Gluconate With Temozolomide in Unmethylated Glioblastoma Multiforme	glioblastoma	125 mg disulfiram is taken orally, twice daily	II	2018	Terminated	The study evaluates the impact of the disulfiram + copper combination when added to standard Temozolomide in the treatment of unmethylated Glioblastoma Multiforme patients.
11.	NCT03151772	Bioavailability of Disulfiram and Metformin in Glioblastomas	glioblastoma	200 mg disulfiram two times daily	I	2018	Terminated	The study analyzes the tumor concentration of preoperatively administered repurposed drugs.
12.	EUCTR2016-004471-46-SE	Study of new compounds in the treatment of brain tumors: drug levels and signs of effect inside the tumor tissue (INSIDE)	glioblastoma	Effervescent disulfiram tablet, 200 mg	III	2017	Not Recruiting	The study investigates the tissue levels and target effect of disulfiram with copper-supplement and metformin.
13.	NCT03034135	Safety, Tolerability and Efficacy of Disulfiram and Copper Gluconate in Recurrent Glioblastoma	glioblastoma	80 mg disulfiram taken orally, three times a day	II	2017	Completed	This study of disulfiram-copper in combination with temozolomide for recurrent glioblastoma will evaluate the antitumor effect in patients who have recurrent glioblastoma. Results: The combination had limited activity for glioblastoma and does not appear to significantly restore temozolomide sensitivity.
14.	NCT02678975 and EudraCT 2016-000167-16	Disulfiram in Recurrent Glioblastoma	glioblastoma	Disulfiram 400 mg daily	II/III	2017	Completed	The study aims to investigate disulfiram and copper-supplement as add-on treatment in glioblastoma patients with recurrence receiving alkylating chemotherapy. Results: The study was closed early due to signs of more adverse events in combination with a very low chance of effect.
15.	CTIS2024-514665-19-00 and EUCTR2016-001386-81-CZ and NCT03323346	Phase II Open-Label Trial of Disulfiram with Copper in Metastatic Breast Cancer	metastatic breast cancer	One pill of disulfiram (Antabus) daily at a dose of 400 mg	II	2017	Recruiting	The study evaluates the efficacy of the treatment by assessing clinical response rate and clinical benefit rate. The aim of the study is to establish clinical evidence for introducing disulfiram and copper as an active therapy for metastatic breast cancer upon failure of conventional systemic and/or locoregional therapies.
16.	NCT02963051	A Phase Ib Study of Intravenous Copper Loading with Oral Disulfiram in Metastatic, Castration Resistant Prostate Cancer	prostate cancer	80 mg disulfiram, three times a day	I	2017	Terminated	The purpose of this study is to determine the safety and optimal dosing of intravenous copper chloride and disulfiram in men with metastatic castrate-resistant prostate cancer.
17.	NCT02770378	A Proof-of-concept Clinical Trial Assessing the Safety of the Coordinated Undermining of Survival Paths by 9 Repurposed Drugs Combined With Metronomic Temozolomide (CUSP9v3 Treatment Protocol) for Recurrent Glioblastoma	glioblastoma	250 mg p.o. once at induction, then twice daily	I/II	2016	Completed	The proof-of-concept clinical trial assesses the safety of the coordinated undermining of survival paths by 9 repurposed drugs (aprepitant, auranofin, captopril, celecoxib, disulfiram, itraconazole, minocycline, ritonavir, and sertraline) combined with metronomic temozolomide for recurrent glioblastoma. Results: The combination has potentially positive effects and, under careful monitoring, is safe.
18.	NCT02715609	Disulfiram/Copper with Concurrent Radiation Therapy and Temozolomide in Patients with Newly Diagnosed Glioblastoma	Glioblastoma	From 250 mg to 500 mg	I/II	2016	Completed	The dose-escalation and dose-expansion study investigates disulfiram/copper with concurrent radiation therapy and temozolomide in patients with newly diagnosed glioblastoma. Results: The maximum tolerated dose of disulfiram in combination with copper, during radiotherapy and temozolomide administration, is 375 mg/day; and the combination demonstrates promising preliminary efficacy for glioblastoma.
19.	NCT02671890	Disulfiram and Chemotherapy in Treating Patients with Refractory Solid Tumors or Metastatic Pancreatic Cancer	metastatic pancreatic adenocarcinoma; refractory malignant solid neoplasm; pancreatic cancer	orally, at the assigned dose level	I	2016	Terminated	The trial studies the side effects and best dose of disulfiram when given together with chemotherapy in treating patients with a solid tumor that does not respond to treatment (refractory) or pancreatic cancer that has spread to other places in the body (metastatic), and to compare whether disulfiram and chemotherapy may reduce tumor-induced muscle loss. The study was terminated due to a lack of funding.
20.	NCT02101008	Disulfiram and Chelated Zinc for the Rx of Disseminated Mets Mel That Has Failed First Line Therapy	metastatic melanoma	250 mg orally per day at bedtime	II	2013	Completed	The study has as a primary objective to determine the response rate associated with the treatment of refractory disseminated malignant melanoma with disulfiram and chelated zinc. Results: The objective was not achieved, adverse events occurred, such as fatigue, confusion, nausea, diarrhea and so forth.
21.	NCT01907165	Disulfiram in Treating Patients With Glioblastoma Multiforme After Radiation Therapy With Temozolomide	glioblastoma	from 500 mg to 1000 mg, taken orally	I	2013	Completed	The trial studies disulfiram in treating patients with glioblastoma multiforme who have completed radiation therapy with temozolomide. Results: The maximum tolerated dose of disulfiram was 500 mg daily in combination with adjuvant temozolmide; and yielded limited proteasome inhibition on peripheral blood cells, which was one of the proposed mechanisms of its antitumor effect.
22.	NCT01118741	Study of Recurrent Prostate Cancer With Rising Prostate Specific Antigen (PSA)	prostate cancer	cohort 1: 250 mg per os daily for 28 days cohort 2: 500 mg per os daily for 28 days	N/A	2010	Completed	The study hypothesizes that disulfiram is a DNA methyltransferase inhibitor and may provide benefit for patients with prostate cancer by restoring tumor suppressor genes. Results: No changes in PSA-based outcomes were observed on this trial, lacking robust activity coupled with the high rates of toxicity
23.	NCT00571116	Disulfiram Plus Arsenic Trioxide In Patients With Metastatic Melanoma and at Least One Prior Systemic Therapy	metastatic melanoma	250 mg per os twice daily	I	2006	Terminated	The trial is studying the side effects and best dose of arsenic trioxide when given together with disulfiram in treating patients with metastatic and progressive melanoma. Terminated because of slow accrual and lack of study funding.
24.	NCT00256230	Disulfiram in Patients With Metastatic Melanoma	metastatic melanoma	250 mg per os twice daily	I/II	2002	Completed	The purpose of the study is to determine the response rate and evaluate the toxicity of disulfiram in the treatment of Stage IV melanoma.

**Infectious Disease**								

1.	ChiCTR2100048035	A clinical study evaluating the effect of disulfiram on the diversity and structure of gut microbiota in healthy volunteers	healthy volunteer	Disulfiram oral, 250 mg daily after dinner	N/A	2021	Pending	The study evaluates the effect of disulfiram on the diversity and structure of gut microbiota in healthy volunteers
2.	NCT04485130	DISulfiram for COVID-19 (DISCO) Trial	COVID-19	1000 mg/day in cohort 1; 2000 mg/day in cohort 2	II	2021	Terminated	The study evaluates disulfiram’s effect on COVID-19 symptom severity, SARS-CoV-2 viral load, and biomarkers of inflammation and pyroptosis (aberrant pro-inflammatory cell death). Results: The study did not accomplish the objectives. The study findings do not support the use of disulfiram in hospitalized patients with moderate COVID-19 in addition to the standard of care.
3.	RBR-5n4htp	New use of a drug named disulfiram for Chagas disease treatment	Chagas disease; American trypanosomiasis	250 and 500 mg/day of disulfiram	I/II	2020	Recruiting	The study aims to assess a combination of drugs for Chagas disease therapy, assuming benznidazol as the drug of choice plus disulfiram as a repositioned drug.
4.	NCT04594343	Clinical Study to Evaluate the Effects of Disulfiram in Patients With Moderate COVID-19	COVID-19	500 mg of disulfiram orally	II	2020	Completed	This clinical trial evaluates the safety, efficacy, and biomarker levels of disulfiram in the treatment of adult subjects hospitalized with moderate COVID-19.
5.	NCT03891667	Disulfiram: A Test of Symptom Reduction Among Patients With Previously Treated Lyme Disease	lyme disease	8 weeks, starting from 250 mg every other day up to 500 mg/daily	I/II	2019	Completed	The study evaluates the safety of disulfiram among patients with post-treatment Lyme disease symptoms. This study did not reach its goal of 24 participants largely due to the impact of the COVID-19 pandemic on the ability to recruit subjects.
6.	NCT03198559	Combination Latency Reversal With High Dose Disulfiram Plus Vorinostat in HIV-infected Individuals on ART	HIV	2 g (4 × 500mg tablets) of disulfiram per day for a total of 28 days	III	2017	Terminated	The purpose of this research is to investigate whether it may be possible to reduce the frequency of latently infected cells in HIV-infected individuals with antiretroviral therapy through pharmacological agents that reverse HIV latency, thereby initiating virus-mediated cell lysis or immune-mediated killing. Early termination of the study led to a small number of participants being analyzed. Therefore, there was insufficient power to measure the effect of the intervention.
7.	NCT01944371	Short-term Disulfiram Administration to Reverse Latent HIV Infection: a Dose Escalation Study	HIV	from 500 mg to 2000 mg disulfiram by mouth per day for 3 days	I/II	2013	Completed	The purpose of this study is to determine the safety, pharmacology, and bioactivity of disulfiram in antiretroviral-treated HIV-infected adults. Results: Disulfiram was well tolerated at all doses. Short-term administration of disulfiram resulted in increases in cell-associated unspliced HIV RNA at all doses, consistent with activating HIV latency.
8.	NCT01286259	Short-term Disulfiram Administration to Accelerate the Decay of the HIV Reservoir in Antiretroviral-treated HIV Infected Individuals	HIV	500 mg disulfiram per day by mouth for 14 days	N/A	2011	Completed	The purpose of this study is to determine whether a two-week course of disulfiram will reduce the HIV-1 latent reservoir in patients on highly active antiretroviral therapy. Results: Disulfiram was well tolerated. The primary outcome was the fold-change, 1.16 infectious units per million cells, after disulfiram administration. The number of participants who had a detectable viral load (>50 copies RNA/mL) was 1 from 16.

**Metabolic Disease**								

1.	NCT05162001	Body Weight Response With Disulfiram in Humans	obesity	Disulfiram 250 mg	I	2021	Unknown	The proof-of-concept study aim to determine if the use of disulfiram for 9 weeks significantly decreases body weight.

**Cognitive disease**								

1.	NCT03212599	Disulfiram as a Modulator of Amyloid Precursor Protein-processing (DIMAP)	Alzheimer’s disease	Disulfiram 1500, 1000, and 500 mg is used for the first three days, and then a dose of 500 mg three times a week	N/A	2013	Completed	The protein ADAM10 represents a promising target for an A-beta peptide prevention strategy. Treatment of human neuronal cells with Disulfiram, a drug which is used in clinical routine for the recrudescence prevention of alcohol dependency, revealed an increased expression of ADAM10. This finding indicates a neuroprotective potential of disulfiram.

**Eye Diseases**								

1.	NCT06319872	The Effects of Disulfiram (Antabuse®) on Visual Acuity in Patients With Retinal Degeneration	Retinal Dystrophies; Age-Related Macular Degeneration; Retinitis Pigmentosa; Stargardt Disease	Oral disulfiram 250 mg/day	I	2025	Not yet recruiting	The study aims to evaluate the impact of oral disulfiram on the vision of patients with retinal degeneration who are being treated with the drug in the management of their concurrent alcohol use disorder.
2.	NCT05626920	Disulfiram for the Treatment of Retinal Degeneration	Inherited Retinal Dystrophy Primarily Involving Sensory Retina	Disulfiram 250 mg	I/II	2024	Recruiting	The cross-over randomized control trial evaluates the retinaldehyde dehydrogenase inhibitor, disulfiram, in improving retinal sensitivity in eyes affected by inherited retinal degeneration.
3.	ISRCTN99747720	A study looking at the mechanism of action of a drug called disulfiram in patients with Ocular Fibrosis in Mucous Membrane Pemphigoid	Ocular Fibrosis; Mucous Membrane Pemphigoid	Single loading dose of 600 mg of oral disulfiram followed by a daily dose of 200 mg	0	2024	Recruiting	The study evaluates the inhibition of ALDH activity in tears to confirm the pharmacological engagement of disulfiram in the eye (reduction in ALDH activity).

**Inflammatory Diseases**								

1.	ISRCTN17596258	AIR-NET- Testing anti-inflammatories for the treatment of bronchiectasis	Bronchiectasis	Disulfiram 200 mg, two tablets taken orally, once daily	II	2024	Recruiting	The study evaluates the effect of a range of interventions (disulfiram, dipyridamole, doxycycline) compared to usual care on the activity of neutrophil elastase in sputum.
2.	JPRN-jRCTs031220648	An exploratory study of the safety and efficacy of disulfiram in progressive fibrosing interstitial lung disease	Progressive Fibrosing Interstitial Lung Disease	0.2 g of disulfiram once a day	II	2023	Recruiting	The study evaluates the safety and efficacy of disulfiram in progressive fibrosing interstitial lung disease.
3.	DRKS00013748	Importance of mechanisms and pathophysiology of cutaneous inflammasome activation in contact dermatitis	Contact Dermatitis	Disulfiram 5% in a base cream for 24 h	N/A	2019	Completed	The study evaluates the effect of two local inflammation inhibitors, disulfiram-cream (already used as oral treatment in patients with contact dermatitis) and mometasone-cream (corticosteroid cream), on the inflammasome. Results: Disulfiram inhibited the SDS-induced eczema *in vivo*. Topical application of disulfiram represents a potential treatment option for irritant contact dermatitis.

**Non-Alcohol Dependence**								

1.	NCT00729300	A Study of the Relationship Between Disulfiram and Cocaine Self-administration.	Cocaine Dependence	250 mg daily	I/II	2006	Completed	The study aims to determine the effects of treatment with disulfiram on cocaine self-administration using a human laboratory model of cocaine self-administration. Results: Disulfiram’s impact on the reinforcing effects of cocaine depends on dose relative to body weight.
2.	NCT00149630	Pharmacogenetics of Disulfiram for Cocaine (Disulfiram)	Cocaine Dependence	250 mg daily	II	2005	Completed	This study evaluates the effectiveness of disulfiram in preventing drug relapse among cocaine and opiate addicts with varying inherited levels of dopamine β-hydroxylase. Results: This study indicates that the dopamine β-hydroxylase genotype of a patient could be used to identify a subset of individuals for which disulfiram treatment might be an effective pharmacotherapy for cocaine dependence.
3.	NCT00580827	Clinical Efficacy of Disulfiram in LAAM-Maintained Cocaine Abusers	Cocaine Dependence	Disulfiram at 62.5 mg/day, disulfiram at 125 mg/day, or disulfiram at 250 mg/day	N/A	2003	Completed	The study assesses the clinical efficacy of disulfiram in LAAM-maintained cocaine abusers.
4.	NCT00913484	Disulfiram for Cocaine Abuse in Buprenorphine Treatment	Cocaine Dependence	Disulfiram 250 mg per day	II	2000	Completed	The study evaluates the efficacy and potential mechanisms of action of disulfiram (versus placebo) for treating cocaine abuse in subjects with concurrent opiate dependence and cocaine abuse or dependence maintained on buprenorphine/naloxone combination. Results: The findings provide limited support for the efficacy of disulfiram for reducing cocaine use and suggest that its mechanism of action may involve the inhibition of dopamine β-hydroxylase.

Abbreviations: ADAM10: A disintegrin and metalloproteinase domain-containing protein 10; ALDH: aldehyde dehydrogenase; CA-19-9: carbohydrate antigen 19-9; COVID19: coronavirus disease 2019; CTC_EMT: circulating tumor cells (CTC) and their epithelial-mesenchymal transition (EMT); CUSP9v3: coordinated undermining of survival paths combining 9 repurposed non-oncological drugs; HER2: human epidermal growth factor receptor 2; HIV: human immunodeficiency virus; LAAM: Levo-alpha-acetylmethadol; PSA: prostate-specific antigen; SDS: sodium dodecyl sulfate.

### Off-label use of disulfiram

This section presents a qualitative synthesis of the main therapeutic areas in which DIS has been investigated for off-label use. Using a structured population, intervention, comparison, and outcome (PICO) framework, the review includes both clinical and translational preclinical , while clearly distinguishing between investigational repositioning and documented off-label use in humans. The number of published studies describing actual off-label use of DIS remains small, limiting the breadth of clinical conclusions. The clinical trials do not represent true off-label use, as they are ethically approved. Their results are extensively discussed by other therapeutic application-oriented reviews, especially in cancer^16^ and infectious diseases.^17^ The following subsections summarize human evidence on the efficacy, safety, and tolerability of DIS in alternative indications beyond alcohol use disorder.

#### Quality assessment of clinical evidence and safety signal summary

Independent of therapeutical applications, a significant number (14 of 44, 31.8%) of the clinical trials investigating DIS did not accomplish their objectives, due: insufficient recruitment (7 trials, 15.9%), inadequate efficacy response (4 trials, 9.1%), unacceptable adverse events (2 trials, 4.5%), and lack of funding (1 trial, 2.3%). However, they offer human data regarding DIS use beyond indication, with clear information on therapeutic application areas, dose, and administration route. The therapeutic areas and the number in each category of the 44 clinical trials conducted in the last decade are: cancer (24), infectious diseases (8), non-alcohol dependence (4), inflammatory diseases (3), eye diseases (3), cognitive diseases (1), metabolic diseases (1) (Table 1). All the clinical trials used oral DIS as tablets or effervescent tablets, with one exception in contact dermatitis, using 5% DIS in a base cream for 24 h (DRKS00013748). The topical application is also a well-known administration route of DIS, authorized in Sweden and Norway for scabies, head lice, and pubic lice treatment, available over-the-counter and sold under the brand name of Tenutex, which is a cutaneous emulsion containing 2% DIS and 22.5% benzyl benzoate as active ingredients. The daily maximum dose of oral DIS, used to treat alcohol dependence, is generally 500 mg, whereas in clinical trials for cancer treatment, a dose range of 240–1000 mg was used, for infectious disease, 250–2000 mg, for cognitive disorders, 500–1500 mg, and in other therapeutic investigations the doses remain below 500 mg. The high failure rate of clinical trials reflects not only challenges in trial design and recruitment but also signals concerning toxicity and efficacy issues that warrant careful evaluation. DIS has experienced declining clinical use in its own indication area due to risks of hepatotoxicity and neurotoxicity, which can lead to serious health complications, rendering DIS uncommon in alcohol cessation therapy.^18^ The tendency of using higher doses in new therapeutic applications of DIS and the adverse events history of the drug uncover the issue of risk-benefit ratio assessment, recently mentioned by Luo J. et al., as a result of Food and Drug Administration (FDA) Adverse Event Reporting System database analysis.^18^ A notable finding from the analyzed reports in this study was the substantial risks linked to off-label use of DIS, such as encephalopathy associated with cancer treatment and Jarisch-Herxheimer reaction in relation to treating Lyme disease.^18^ The product information table of DIS enumerates common adverse effects, including headache, drowsiness, fatigue, and a metallic or garlic-like taste, and severe reactions such as neurotoxicity and hepatotoxicity, which are more frequent at higher doses or when combined with other medications.^18^ Luo et al. identified unlabeled adverse events associated with the drug, including delirium, vocal cord paralysis, and ST-segment depression on electrocardiogram.^18^ According to this study's outcomes, from 2014 to 2020, the number of DIS adverse event reports generally declined, while in 2021, there was a significant increase, due to trials in antitumor therapy,^18^ which could be interpreted as another impact of COVID-19-induced drug repositioning. Limited clinical evidence supports routine off-label DIS use beyond approved indications; observed benefits are modest and inconsistent, while safety risks are substantial and underexplored at higher doses.

#### Cancer

Data from ongoing or completed clinical trials are the determinants of off-label uses, particularly regarding toxicity and safety. The phase I/II clinical trials (NCT01907165, NCT02715609) investigating DIS for glioblastoma treatment, conducted by Huang J. et al., evaluated the safety and preliminary efficacy of DIS combined with copper and with concurrent standard radio- and chemotherapy.^19^^,^^20^^,^^21^ The studies concluded that DIS can be safely administered alongside temozolomide, though it may trigger reversible neurological side effects.^19^ Toxicity profiles and pharmacodynamic responses for DIS remained comparable whether copper was used concurrently or not.^21^ The maximum tolerated dose of DIS combined with radiotherapy and temozolomide stands at 375 mg per day, with DIS plus copper demonstrating limited efficacy in most patients but notable promise in BRAF-mutant glioblastoma cases.^20^ Another clinical trial (NCT02770378) investigated a treatment regimen with 9 different drugs [Coordinated Undermining of Survival Paths combining 9 repurposed non-oncological drugs (CUSP9v3): aprepitant, artesunate, auranofin, captopril, celecoxib, DIS, itraconazole, sertraline, and ritonavir] in addition to low-dose metronomic temozolomide concluding that CUSP9v3 can be safely administered in patients with recurrent glioblastoma under careful monitoring, and the most common adverse events identified were nausea, headache, fatigue, diarrhea, and ataxia.^22^^,^^23^ A case study reported a 37-year-old male with a left parieto-occipital multifocal glioblastoma, who underwent partial resection of the tumor, whereafter he received DIS (250 mg x 1, p.o.) and copper-gluconate (3 mg x2, p.o) along with the standard radio-chemotherapy, exhibiting remarkably increased progression-free and overall survival.^24^ In spite of low-dose DIS, the treatment had to be stopped twice for 15 days each time due to the transient elevation of transaminase levels.^24^ Another case report describes the emergence of posterior reversible encephalopathy syndrome following alcohol-free DIS exposure, underscoring the need for consultation-liaison psychiatrists to maintain a high index of suspicion for adverse drug reactions associated with off-label use for antineoplastic (metastatic melanoma) purposes.^25^ In contrast, a nationwide epidemiological study revealed that patients who continuously used DIS have a lower risk of death from cancer compared to those who stopped using the drug at their diagnosis.^26^

#### Infectious diseases

Case reports represent a valuable method for highlighting the potential efficacy of novel therapeutic interventions.^27^ In 2019, three clinical instances were published, introducing DIS as a potential treatment for chronic, relapsing tick-borne illnesses,^28^ unintended initiating the off-label use of DIS in Lyme disease. Applying a retrospective review, the clinical understanding has expanded through a larger population, 67 individuals were treated with DIS by 2020, falling into a “high-dose” group (≥4 mg/kg/day) or a “low-dose” group (<4 mg/kg/day).^29^ According to this study, high-dose adverse events included fatigue, psychiatric symptoms, peripheral neuropathy, and mild-moderate liver enzyme elevations. Despite higher adverse reaction risk, high-dose patients were significantly more likely to attain enduring remission.^29^ The French Federation Against Tick-Borne Diseases (FFMVT, its French acronym) was alerted to severe and persistent toxic reactions in a patient with late disseminated Lyme disease after taking DIS.^27^ In response, FFMVT issued a national call to identify other patients in France who received similar treatment and to gather reports on any benefits or side effects. Of the 16 respondents, 13 reported DIS-induced adverse effects, predominantly affecting the nervous system (e.g., neuropathies, headaches, dizziness, and cognitive impairment, including concentration and expressive difficulties, sleep disturbances, exacerbated generalized pain, and increased fatigue).^27^ Several patients also noted more specific aggravations, such as osteoarticular pain, nausea, or gastrointestinal disorders.^27^ Certain DIS toxicities in patients with Lyme may stem from high initial doses akin to those for alcohol dependence, or from Jarisch-Herxheimer reactions due to DIS-induced *Borrelia burgdorferi* die-off. However, patients with prior Herxheimer episodes reported distinct reaction profiles with DIS.^27^ The pilot study evaluating DIS safety, tolerability, and clinical response in Lyme disease treatment (NCT03891667) highlighted risks, particularly at higher doses, where tolerability was poor. Although >50% of participants reported clinical benefit, the small sample size and lack of placebo control preclude definitive conclusions on DIS efficacy for persistent post-Lyme symptoms.^30^ Clinicians now emphasize individualized dosing and close monitoring to mitigate side effects.^31^

In contrast to Lyme disease, no documented cases of off-label DIS use for human immunodeficiency virus (HIV) were identified in this research outside controlled clinical trials. Combination antiretroviral therapy (ART) delivers substantial health benefits to individuals with HIV but fails to eradicate the virus. HIV persists mainly by establishing latency in long-lived memory CD4^+^ T-cells. Developing a safe, scalable cure remains a critical public health goal to alleviate the personal and societal burdens of lifelong infection and treatment. Preclinical research identified DIS as capable of reactivating HIV transcription in a primary CD4^+^ T cell latency model. A follow-up pilot trial administered 500 mg daily for 14 days to 16 ART-suppressed adults (NCT00718003). Recently, a short-term dose-escalation study (NCT01944371) confirmed DIS’s safety and tolerability, showing elevated plasma HIV RNA at higher doses.^32^

The studies evaluating DIS’s effect on COVID-19 symptom severity, SARS-CoV-2 viral load, and biomarker levels were closed with unaccomplished objectives (NCT04485130, NCT04594343).

#### Inflammatory diseases

DIS ameliorates nonalcoholic steatohepatitis (NASH) through gut microbiota modulation. DIS inhibits Clostridium growth, reducing Clostridium-mediated 7α-dehydroxylation and secondary bile acid biosynthesis, thereby activating hepatic farnesoid X receptor (FXR) signaling. A self-controlled clinical trial (ChiCTR2100048035) in 23 healthy volunteers receiving DIS 250 mg daily for 7 days demonstrated reduced Clostridium 7α-dehydroxylation activity, with favorable tolerability and no serious adverse events. Fecal microbiota transplantation from DIS-treated humans into germ-free mice confirmed NASH improvement.^33^

Another clinical study (DRKS00013748) evaluated DIS cream, previously used orally for contact dermatitis, and assumed that topical application represents a promising therapeutic option for irritant contact dermatitis.

#### Cognitive diseases

In a natural clinical setting of DIS, used in alcohol dependent patients, the evaluation of its potential to upregulate a disintegrin and metalloproteinase domain-containing protein 10 (ADAM10) expression in human peripheral blood cells, also including a healthy control group without DIS treatment, to analyze ADAM10 expression (NCT03212599). ADAM10 functions as a metalloproteinase that cleaves the amyloid precursor protein via alpha-secretase activity in neurons. This enzymatic process yields a neuroprotective amyloid precursor protein cleavage product (sAPPα) while preventing the formation of amyloidogenic Aβ peptides, key pathological features of Alzheimer’s disease. Whereas most healthy controls displayed an unaltered ADAM10 expression, the majority of DIS-treated patients demonstrated elevated ADAM10 mRNA. The induction of ADAM10 in humans in peripheral tissue by DIS treatment is thus feasible, but efficacy and safety were not tested in patients with Alzheimer’s disease.^34^ According to the studies’ methodology, this experiment does not reflect a typical off-label use, but the promising results potentially predict such an application, even though the authors emphasized the lack of safety and toxicity studies.

#### Non-alcohol dependence

DIS represents effective pharmacotherapy for cocaine addiction, potentially acting via copper chelation and inhibition of dopamine β-hydroxylase, the enzyme that converts dopamine to norepinephrine. Off-label prescribing persists based on this evidence, the findings of clinical trials (NCT00729300, NCT00149630, NCT00913484) provide limited support for the efficacy of DIS for reducing cocaine dependence.^35^^,^^36^^,^^37^

#### Other diseases

A case report details an industrial technician with occupational nickel carbonyl exposure. Treatment initiated with DIS (1 g orally, followed by 500 mg 6 h later, then 250 mg twice daily for 5 days) alongside prednisone (60 mg orally for 5 days). Forty-eight hours post-initiation, the patient exhibited exacerbated respiratory distress, with oxygen saturation declining to 85% despite 2 days of oral steroids, necessitating hospitalization; he received prednisone (60 mg/day orally), 4 L nasal oxygen, and DIS (500 mg twice daily), achieving discharge on day 7 post-exposure while continuing both agents. DIS administration was off-label, guided by an established corporate protocol.^38^

### Repurposed use of disulfiram

While the actual off-label human use remains surprisingly limited, the preclinical literature on DIS is extensive and mechanistically diverse. Research on its mechanism of action shows that the DIS and copper complex, bis(diethyldithiocarbamate)-copper [Cu(DDC)_2_], could be the decisive metabolite for tumor-suppressing effects.^10^ However, the combination of DIS and copper does not have the identical molecular mechanisms to Cu(DDC)_2_ nor the simple addition effect of DIS and copper.^10^ On the other hand, drug repositioning excludes any structural changes to the drug. It repurposes existing biological properties—for which the drug was already approved (potentially with a different formulation, dose, or administration route) or the side properties of a drug that are responsible for its adverse effects.^6^ Therefore, using DIS repositioning terminology in this context can be misleading, especially when it comes to the pharmaceutical technology development of a metabolite, copper-complex,^39^^,^^40^^,^^41^^,^^42^^,^^43^^,^^44^^,^^45^^,^^46^^,^^47^^,^^48^^,^^49^^,^^50^^,^^51^ or analogs,^52^ which are new chemical entities rather than drug repurposing, with different toxicity and safety profiles. Whereas in clinical trials solely DIS is applied with or without copper co-administration, in primary cancer research, a fusion of the drug and its metabolite is traceable both in pharmacology and drug development studies, with DIS interpreted as a prodrug of Cu(DDC)_2_. This integration of the drug and its metabolite is not observed in other application fields, such as infectious, inflammatory diseases, and metabolic disorders. This section summarizes the mechanism of actions of DIS in repositioning areas, identified in the last decade, and also emphasizes the importance of formulation strategies. The pharmacokinetics of DIS—both in its primary indication and beyond—falls outside the scope of this study, as in-depth reviews specific to those treatment areas already address it.^9^^,^^53^ The following subsections focus on mechanistic findings across different therapeutic areas and highlight drug development perspectives for DIS.

#### Mechanistic studies

##### Cancer

A growing body of preclinical research supports the repositioning of DIS as a promising anticancer agent across a broad range of malignancies, with enhanced antineoplastic activity upon copper combination. The mechanistic studies described the following pathways:

**Reactive oxygen species generation:** The primary anticancer mechanism of action described was the generation of reactive oxygen species (ROS), which damage DNA, protein, and lipids, ultimately driving cells toward apoptosis.^54^ DIS targets cancer cells in a copper-dependent manner through two complementary effects: (1) instant short-term killing via ROS generated by the reaction of DIS and copper, and (2) delayed, long-lasting cytotoxicity from the end product, Cu(DDC)_2_.^54^ *In vivo*, DIS is rapidly reduced to diethyldithiocarbamate (DDC) in the bloodstream; DDC is highly unstable and quickly converts to irreversible downstream metabolites.^54^ Given the extremely short half-lives of both DIS and DDC in circulation, no ROS and Cu(DDC)_2_ forms when oral DIS and copper are administered separately to patients—explaining the discrepancy between *in vitro* experiments and clinical outcomes.^54^ In contrast with DIS, another of its metabolites, Cu(DDC)_2_ is a stable compound with a serum half-life exceeding 4 h, and may follow the rule of conventional anticancer drugs, interfering with vital molecular pathways in cancer cells to induce apoptosis.^54^ The complex accumulates intracellularly over prolonged periods. This triggers cellular morphological changes, elevates ROS levels, arrests the cell cycle in G0/G1 phase, and ultimately induces apoptosis.^55^ Moreover, the acidic microenvironment, a common feature of many solid tumor tissues, could promote intracellular Cu(DDC)_2_ via two complementary mechanisms, without altering its uptake (1) it enhances transmembrane transport of dithiocarb by converting its ionic form to a neutral molecular state; and (2) it boosts Cu^2+^ uptake by activating the copper transporter hCTR1, ultimately enriching Cu(DDC)_2_ levels.^56^ An insufficiency of metallothioneins sensitizes cancer cells toward the copper complex, suggesting a potential predictive biomarker for DIS treatment efficacy.^57^

**Apoptosis and autophagy:** In addition to ROS generation,^58^ several mechanisms have been reported for the induction of apoptosis by DIS in cancer cells, including the inhibition of proteasome activity,^59^^,^^60^ regulation of transcription factors [e.g., nuclear factor-kappa B (NF-κB)],^61^^,^^62^^,^^63^^,^^64^ activation of the stress-related c-Jun N-terminal kinases (JNK) signaling pathway^65^^,^^66^ and activation of the mitochondria-related intrinsic apoptotic pathway, reflected by loss of mitochondria membrane potential, down-regulation of anti-apoptotic B-cell lymphoma 2 (Bcl-2) family proteins [e.g., Bcl-2 and B-cell lymphoma-extra large (Bcl-xL)], following caspase-3 activation and poly ADP ribose polymerase (PARP) degradation.^67^^,^^68^^,^^69^ The copper-complex also triggers endoplasmic reticulum (ER) stress via the inositol-requiring enzyme 1α - X-box binding protein 1 (IRE1α-XBP1) pathway, which partly drives autophagy-dependent apoptosis in cancer cells.^70^ In addition, the treatment shows potent cytotoxic effects via ER stress-relevant binding immunoglobulin heavy chain protein (Bip) mediated apoptosis and suppressing autophagy.^69^ DIS induces G1 cell-cycle arrest, apoptosis, and autophagic cell death via blockade of AKR strain transforming protein kinase (Akt) and activation of Forkhead box O (FOXO) transcription factors.^71^ DIS with copper disrupts mitochondrial homeostasis, expands the free iron pool, boosts lipid peroxidation, and ultimately triggers ferroptotic cell death,^72^^,^^73^ which led to the reduced expression of ferredoxin 1 and loss of Fe-S cluster proteins (biomarkers of cuproptosis).^74^^,^^75^ The drug triggers excessive autophagy via oxidative phosphorylation (OXPHOS) deficiency. Specifically, optic atrophy protein 1 (OPA1) degradation disrupts cristae structure, sharply reducing mitochondrial respiration and ATP production. This energy deprivation activates adenosine monophosphate-activated protein kinase (AMPK), driving autophagy.^76^

**Chemotherapy and immunotherapy sensitizing effects:** DIS enhances the efficacy of DNA-damaging agents by suppressing DNA repair pathways. Notably, DIS directly inhibits O^6^-methylguanine-DNA methyltransferase (MGMT) expression—an enzyme that removes O^6^-alkyl groups from guanine to enable error-free DNA replication. However, this MGMT reduction requires significantly higher DIS concentrations.^59^ The DIS and copper combination robustly activates cancer cell-intrinsic cyclic GMP-AMP synthase-stimulator of interferon genes (cGAS-STING) signaling. It induces mitochondrial/nuclear DNA damage and cytosolic double-stranded DNA release via excessive ROS generation—triggering innate immunity, boosting antitumor effects, enhancing CD8^+^ T cell and natural killer (NK) cell infiltration, and potentiating programmed cell death protein 1 (PD-1) checkpoint blockade efficacy in murine models.^77^

**Radiotherapy sensitizing effect:** DIS combined with copper sensitizes ionizing radiation-resistant cancer stem cells, due to elevated ROS level, amplifying radiation damage, and by the activation of the IRE1α/XBP1 signaling axis.^78^ *In vitro* and *in vivo* studies showed that the copper-complex radiosensitizes ALDH1-positive carcinoma cells by inhibiting ALDH1 and downregulating the phosphoinositide 3-kinase (PI3K)/Akt pathway, particularly enhancing the radiosensitivity of tumor-initiating cells.^79^ As ALDH serves as a CSC biomarker, DIS has been proposed as a promising option.^80^ Yet, DIS is essential for heightened cytotoxicity, and ALDH inhibition alone cannot fully explain its therapeutic effects *in vitro* and *in vivo.*^59^ Moreover, this theory seems to be failing, as DIS does not directly suppress ALDH across human cell types; the inhibition is attributed to its *in vivo* metabolite S-methyl-N,N-diethylthiocarbamate-sulfoxide, which does not affect the viability of cancer cells. DIS anticancer effects instead arise from its copper-containing metabolite, which forms spontaneously *in vitro* and *in vivo*, that induces nuclear protein localization 4 (NPL4) aggregation, disrupting the p97/valosin-containing protein (VCP) segregase.^81^ In addition, DIS modulated cell cycle distribution in cancer stem cell cultures and dramatically decreased clonogenic survival independently of ALDH1A3 expression.^82^

**NPL4-p97/VCP pathway:** DIS`s definitive antineoplastic mechanism of action, identified over the past decade, involves the molecular target NPL4, which is an adapter for the p97/VCP segregase and is essential for protein turnover, playing a role in several regulatory and stress-responsive cellular pathways.^26^ DIS’s cytotoxic activity stems from its rapid metabolism to Cu(DDC)_2_, which preferentially accumulates in tumors. Intracellular Cu(DDC)_2_ binds NPL4, inducing aggregation that disrupts the essential p97-NPL4-UFD1 segregase pathway. This triggers a multifaceted cellular stress response culminating in cell death,^26^ interferes with DNA replication, slowing fork progression and promoting single-stranded DNA accumulation, inducing replication stress, S-phase-specific DNA damage, and activation of homologous recombination repair.^83^ The Cu(DDC)_2_-triggered replication stress is seriously impaired due to the concomitant malfunction of the ATR-interacting protein - ataxia-telangiectasia and Rad3-related protein kinase-checkpoint kinase 1 (ATRIP-ATR-CHK1) signaling pathway that reflects an unorthodox checkpoint silencing mode through Ataxia telangiectasia and Rad3-related kinase sequestration within the complex-evoked NPL4 protein aggregates.^83^ In diverse human cancer cell models, Cu(DDC)_2_ rapidly triggers integrated stress response (ISR)–mediated translational arrest, followed by nucleolar stress. Cu(DDC)_2_ sequesters p53 in NPL4-rich aggregates, elevating p53 levels while functionally inhibiting it, consistent with p53-independent cell death.^84^ Transcriptomic profiling further reveals the pro-survival activation of ribosomal biogenesis (RiBi) and autophagy pathways during prolonged exposure, suggesting adaptive feedback responses.^84^ On the other hand, DIS directly inhibits matrix metalloproteinase (MMP) enzymes by quenching their zinc cofactor, thereby overcoming tissue inhibitor 2 (TIMP2)-mediated activation of pro-MMP2. MMPs and their TIMPs regulate extracellular matrix degradation, influencing processes such as tissue remodeling and cell signaling. MMPs play a key role in cancer progression and invasion; their downregulation reduces cell invasiveness.^60^ Reports link p97 overexpression to carcinoma progression and metastasis, underscoring DIS’s adjuvant potential in metastatic cancers. Its metabolite, Cu(DDC)_2_, binds NPL4 to rapidly induce protein aggregates, crippling the p97-NPL4-ubiquitin fusion degradation 1 (UFD1) segregase pathway. This unleashes a severe cellular phenotype, heat shock response (HSR), and ultimate cell death.^26^

**Other novel targets of disulfiram:** DIS was also reported as one of the first phosphoglycerate dehydrogenase (PHGDH) inhibitors, a tetrameric enzyme catalyzing the initial step of the serine synthetic pathway that is highly expressed in numerous cancer types. DIS disrupts the active tetrameric form of the enzyme through specific cysteine oxidation (involving the cysteine 116 residue), leading to a significant decrease in tumor proliferation. Thus, the non-metabolized circulating DIS could also provide anticancer activity, but through a completely different mechanism of action involving PHGDH inhibition.^85^ Antimetastatic potential of DIS is attributed to the inhibition of cyclooxygenase-2 (COX2), which is an important inflammatory marker, and inflammation is known to be closely associated with tumor development, metastasis, and invasion.^60^ DIS modulates endogenous plasma membrane-associated carbonic anhydrases 12 (CA12) and its associated transporter anion exchanger 2 (AE2) expression while attenuating cancer cell migration and invasion through reduced AE2 activity and levels.^86^ In addition to the aforementioned targets, DIS exerts anti-oncogenic effects by triggering the degradation of the oncoprotein MLL, e.g., MLL1 drives tumor growth and angiogenesis across multiple cancer types; its knockdown potently suppresses *in vivo* tumor progression.^87^ Another novel insight into DIS’s anticancer effects involves its direct activation of lymphocyte-specific protein tyrosine kinase (LCK)-mediated T cell receptor (TCR) signaling, which potently induces antitumor immunity independent of copper ion.^88^ Mechanistically, DIS covalently binds to cysteine 20/cysteine 23 residues of LCK and enhances its tyrosine 394 phosphorylation, thereby promoting LCK kinase activity and boosting effector T cell function, interleukin-2 production, metabolic reprogramming, and proliferation.^88^ Similarly, lysine acetyltransferase 2A (KAT2A)^89^ and deamidated triosephosphate isomerase (dTPI) were introduced as another target of DIS.^90^ Ubiquitin-specific proteases (USPs), featuring a highly conserved zinc-finger subdomain, represent promising anticancer targets, as their upregulation occurs across diverse tumors. Leveraging the metal-chelating properties of DIS, it acts as a competitive inhibitor of USP2 and USP21, two critical tumor-associated deubiquitinases.^91^

##### Infectious diseases

Several studies have explored DIS’s antimicrobial properties *in vitro* and *in vivo*, revealing activity against a broad range of pathogens, including viruses, bacteria, fungi, and parasites.

**Viruses:** HIV persists in a reservoir of latently infected CD4^+^ T cells that do not express viral proteins and appear indistinguishable from uninfected cells. One promising HIV cure strategy, “shock and kill,” seeks to reactivate latent HIV, thereby triggering cytotoxic T cell responses to eliminate infected cells. DIS reactivates HIV transcription in latently infected cell lines by depleting phosphatase and tensin homolog (PTEN), which activates protein kinase B (PKB/Akt) signaling and boosts downstream HIV gene expression.^32^^,^^92^ Recently, DIS was confirmed to be potent SARS-CoV-2 main proteases M^pro^ and PL^pro^ inhibitor, but also targets other SARS-CoV-2 proteins such as nsp13 and nsp14 and is even an inhibitor of pyroptosis via inhibition of gasdermin D.^93^^,^^94^^,^^95^^,^^96^ Moreover, it disrupts the interaction between the viral spike protein and the host Angiotensin-converting enzyme 2 (ACE2) receptor, preventing cell entry across variants.^97^ Ultimately, the inhibitory potential of DIS on M^pro^ and PL^pro^ was contradictory.^98^^,^^99^

**Bacteria:** DIS, an electrophilic compound, readily forms disulfides with thiol-bearing molecules. Bacteria harbor diverse intracellular thiols, including cofactors (e.g., coenzyme A), metabolites (e.g., glutathione, mycothiol, and bacillithiol), and enzymes (e.g., thioredoxin), that DIS can modify via thiol-disulfide exchange, triggering antimicrobial effects.^100^ Along with this, DIS shows promise against Lyme disease by targeting persistent forms of *Borrelia burgdorferi*, the causative bacterium. It disrupts bacterial survival through two key chemical mechanisms: (1) thiol-disulfide exchange with bacterial cofactors and enzymes, which depletes glutathione and halts growth, especially in stationary-phase persisters; and (2) chelation of copper ions essential for bacterial enzymes and oxygen transport.^29^^,^^101^ DIS inhibits *Staphylococcus aureus* growth by disrupting central glucose catabolism and inducing redox imbalance (e.g., oxidative stress), and by chelating metal ions, antagonizing the respiratory chain, ultimately blocking cell replication.^102^ Initial growth deceleration stems from rapid DIS cleavage by cellular thiols in proteins, metabolites, and cofactors, abruptly halting metabolism. Cleavage yields mixed disulfides that undergo further thiol-disulfide exchange (e.g., with glutathione), producing disulfides and DDC.^100^ Exogenous glutathione raises minimal inhibitory concentrations 2-fold for DIS,^100^ explaining its limited activity against thiol-rich Gram-negatives versus Gram-positives.^100^^,^^103^^,^^104^^,^^105^^,^^106^^,^^107^^,^^108^ However, DIS prevents the antibiotic-resistant Gram-negative, *Helicobacter pylori* strain from killing human gastric cells, by inhibiting the activity of bacterial ureases.^109^

**Fungi**: DIS’s antifungal effects arise from inactivating key enzymes, including ALDH, urease (URE), and carbamate kinase (CK). In *Pythium insidiosum*, DIS binds the oomycete’s putative ALDH and URE at low energy, impairing their activities.^110^ DIS disrupts key fungal cell processes (metabolism, replication, and membrane transport), exhibiting potent antifungal activity against *Candida* and *Cryptococcus* species by inhibiting both growth and biofilm formation.^111^^,^^112^^,^^113^

**Parasites**: DIS eradicates *Giardia lamblia* trophozoites by inactivating carbamate kinase via active-site cysteine modification. As a thiol-reactive agent, it also targets *G. lamblia* triosephosphate isomerase at cysteine 222 (species-specific, sparing the human homolog), causing minor conformational changes but substantially reducing enzyme stability.^114^ DIS potently inhibits *Cryptosporidium parvum* by irreversibly targeting inosine 5′-monophosphate dehydrogenase (IMPDH), an essential enzyme in guanine nucleotide biosynthesis. IMPDH disruption offers therapeutic potential across anticancer, immunosuppressive, antiviral, and antimicrobial applications.^115^ In addition, the drug effectively targets the tegument-expressed ALDH SmALDH_312, demonstrating potent activity against *Schistosoma mansoni*.^116^

##### Inflammatory diseases

By targeting gasdermin D (GSDMD)-driven inflammation, DIS offers promise for repurposing against diverse inflammatory diseases, as it selectively inhibits pore formation by GSDMD, sparing other gasdermin family members. Blocks pyroptosis and cytokine release in cells and prevents lipopolysaccharide (LPS)-induced septic death in mice. At nanomolar concentrations, DIS covalently modifies cysteine 191 (human GSDMD) or cysteine 192 (mouse GSDMD). This permits upstream processing of c) and GSDMD but blocks pore formation, thereby preventing IL-1β release and pyroptosis.^117^

DIS inhibits oxidative stress and Nod-like receptor protein 3 (NLRP3) inflammasome activation, thus preventing LPS-induced cardiac injury.^118^ DIS suppresses ferroptosis by modifying cysteine residues on glutathione peroxidase 4 (GPX4), preventing its chaperone-mediated autophagic degradation. This stabilizes GPX4, enabling lipid peroxide detoxification and cytoprotection. The mechanism offers a novel approach to GPX4 regulation, with DIS showing repurposing potential in ferroptosis-driven diseases, as validated in the acute liver injury model.^119^

DIS reduces IL-12/23 (p40) production of dendritic cells; it can be a treatment option for autoimmune inflammatory diseases.^120^

It inhibits macrophage infiltration, likely via FROUNT suppression, an intracellular regulator of chemokine receptors, chemokine receptor 2 (CCR2)/C-C motif chemokine receptor 5 (CCR5) signaling, and attenuates bleomycin-induced lung fibrosis.^121^ The drug attenuates paraquat-induced pulmonary fibrosis by suppressing inflammation, collagen accumulation, and zinc finger E-box binding homeobox 1 (Zeb1)-mediated profibrotic signaling, positioning it as a promising repurposed therapy for idiopathic pulmonary fibrosis.^122^ In addition, it shows promise for treating this by targeting the COX-2/prostaglandin E2 pathway.^123^

##### Cognitive diseases

DIS may influence Alzheimer’s disease pathology as an ADAM10 gene expression enhancer.^34^

##### Metabolic diseases

DIS has shown promising metabolic effects in various preclinical models of diet-induced obesity and metabolic syndrome.

DIS improves glucose tolerance, insulin sensitivity, and multi-organ metabolic function in obesity models. Integrated liver transcriptomic/proteomic analyses reveal shared signatures in lipid/energy metabolism, redox balance, detoxification, and potent autophagy activation.^124^^,^^125^ It potently inhibits ALDH1A1 (more than ALDH2), which converts retinaldehyde to retinoic acid and is overexpressed in obese individuals; ALDH1A1 disruption confers diet-induced obesity resistance in rodents and reduces fat mass in mice/dogs without viability defects.^126^

DIS enhances oxidative phosphorylation-dependent growth in diverse yeast models of mitochondrial diseases, spanning defects in ATP synthase, complexes III/IV, cardiolipin remodeling, and mitochondrial genome maintenance/translation. The compound also benefits patient-derived cells from Barth and Mitochondrial Encephalopathy, Lactic Acidosis, and Stroke-like episodes (MELAS) syndromes, linked to impaired cardiolipin remodeling and intramitochondrial protein synthesis.^127^

##### Other diseases

DIS enhances μ-opioid receptor G-protein signaling, which could, in part, contribute to the augmentation of morphine antinociception and abolition of morphine tolerance development.^128^ The hypothesis on DIS potential to abrogate morphine tolerance is related to the off-label drug use, as DIS was shown to produce clinically relevant enhancement of stimulation-induced analgesia in refractory pain patients that was insensitive to the development of tolerance for a period of 10 months.^129^

#### Drug formulation strategies

There is a growing consensus among researchers that DIS repositioning is feasible through a dual approach: in-depth pharmacological studies and extensive pharmaceutical technology investigations. Despite DIS’s established clinical efficacy in oral alcoholism therapy, its *in vivo* repositioning performance remains suboptimal due to rapid hepatic metabolism and deficient target tissue delivery, which could be overcome by developing novel drug delivery systems.^10^

The pharmaceutical landscape has undergone a paradigm shift with the advent of nanotechnology, holding the potential to overcome the limitations of traditional drug delivery systems,^130^ therefore, it was expected to be the key formulation strategy in the case of DIS repositioning (Table 2). Nanomedicines offer improved bioavailability, reduced dosing frequency and side effects, and enable targeted therapy, making them highly attractive for the treatment of complex diseases such as cancer, infectious, inflammatory diseases, and neurological disorders.^130^ However, this field faces various challenges, such as concerns over safety and toxicity, regulatory hurdles, manufacturing difficulties, and scalability.^130^

**Table 2:** Drug delivery systems of repurposing disulfiram in the last decade (2015–2025)

No.	Name of the drug delivery system	Drug development objective	Technology	Drug carrier	DIS content	Dosage form	Therapeutical application	Reference
1.	Disulfiram-loaded poly-[lactide-co-glycolic acid] nanoparticles (PLGA)	A drug that is minimally soluble in water and is known to have significant side effects may be loaded onto nanoparticles that are designed for maximum drug entrapment and more target-oriented drug delivery.	nanotechnology	• Polysorbate 80 • PLGA	24 μg of DIS entrapped per mg of nanoparticles	N/A	Cancer	Hoda et al.^60^
2.	Poly lactic-co-glycolic acid (PLGA) encapsulated disulfiram nanoparticles	The purpose of this study was to develop a long-circulating poly lactic-co-glycolic acid (PLGA) encapsulated disulfiram (DS-PLGA) to achieve longer circulating time and improve the anticancer efficacy of the drug.	nanotechnology	• PLGA	Drug loading content of nanoparticles is: 27.67 ± 3.47%; and encapsulation efficiency is: 78.92 ± 2.16%	Intravenously applied for mice	Cancer	Wang et al.^131^
3.	Passively-targeted disulfiram-nanoparticles (DISNPs) using biodegradable monomethoxy (polyethylene glycol) d,l-lactic-co-glycolic acid (mPEG-PLGA) matrix	The passively targeted disulfiram nanoparticles, which overcome the drug`s instability and low therapeutic efficacy, can affect multiple targets, trigger potent anticancer effects, and offer a sustained drug supply for brain cancer treatment through enhanced permeability and retention.	nanotechnology	• mPEG • PLGA	The drug loading and encapsulation efficiency were determined to be 18.47% and 92.1%.	Intravenously applied to mice	Cancer	Madala et al.^132^
4.	Nanostructured lipid carriers (NLC) encapsulated disulfiram modified with d-α-tocopheryl polyethylene glycol 1000 succinate (vitamin E-TPGS)	PEGylated nanostructured lipid carriers were introduced to assure an efficient delivery of disulfiram to enhance its application and effectiveness in clinical settings, and thus, increase its stability and bioavailability *in vivo.*	nanotechnology	• solid lipid (Precirol ATO5) • liquid lipid (Labrafac Lipophile WL1349) • soy lecithin • vitamin E-TPGS	Encapsulation efficacy: 80.7 ± 2.68%	Intravenously applied to mice	Cancer	Banerjee et al.^133^
5.	Poly (L-lactic acid) (PLGA) based disulfiram-loaded nanoparticles	Nanoparticles can enhance the intratumoral accumulation of the drug and are expected to enhance permeability and retention, thus strengthening the cancer-specific efficacy of disulfiram. Moreover, nanoformulation is also promising in allowing labeling for specific uptake by cancer cells.	nanotechnology	• PLGA	Drug loading content of nanoparticles is: 11.18 ± 0.42%	Intravenously applied to mice	Cancer	Kita et al. ^134^
6.	Whey protein isolate (WPI) stabilized disulfiram nanosuspension	Drug stabilization: The main functional property of whey protein is as a vehicle for drug delivery systems controlled by the WPI. The presence of amino acids and disulfide bonds inside the protein is mainly responsible for the stabilizing ability of the WPI.	nanotechnology	• WPI	Drug loading content of nanoparticles is: 5.28 ± 1.8%, and encapsulation efficiency is: 87.32 ± 2.6%	N/A	Cancer	Farooq et al.^135^
7.	Soybean lecithin-stabilized disulfiram nanosuspensions	Although disulfiram exhibits significant antitumor activity, it is greatly unstable in an acidic gastric environment. Disulfiram is rapidly degraded in the bloodstream by glutathione reductase, with a half-life of 4 min. This instability hindered the application of disulfiram in the clinic. The present study prepared novel disulfiram nanosuspensions with high drug-loading content using safe nanocarriers.	nanotechnology	• soybean lecithin	Drug loading content: 44.36 ± 1.09%	Intravenously and per os applied for mice	Cancer	Li et al.^136^
8.	Sub-micronized products of disulfiram fabricated by using the rapid expansion of supercritical solutions (RESS) process and improved by coating with polyvinylpyrrolidone (PVP) and methoxy *b*-poly(_L_-lactide) 2000- poly(ethylene glycol) 2000 (mPLLA-PEG) solution using the RESS-based processes	Disulfiram, a copper-dependent antitumor drug, suffers from multiple shortcomings of poor aqueous solubility and instability in the physiological environment, which often hinder its clinical applicability. RESS process can enhance the dissolution rate as well as the substantial antitumor efficiency of nano-sized drug.	nanotechnology	• supercritical carbon dioxide • PVP • mPLLA-PEG	The drug content in the nanoparticles was nearly 100%	N/A	Cancer	Tang et al. ^137^
9.	Plate-like alginate (ALG) microparticles loaded with disulfiram and superparamagnetic iron oxide (SPIO)	The original formulation was designed for use via oral administration, which is not suitable to be given by a direct spray on the affected area. Therefore, the study designed ALG microparticles loaded with disulfiram and superparamagnetic iron oxide (cross-linking disulfiram/SPIO/ALG MPs), which have great potential application for inhibiting the growth of ovarian cancer simultaneously via two treatments, i.e., chemotherapy and hyperthermia.	microtechnology	• alginate • SPIO • calcium chloride	Encapsulation efficacy: 98.89%	Intraperitoneally applied for mice	Cancer	Bai et al.^138^
10.	Folic acid-modified disulfiram/Zn-metal organic framework (IRMOF3) nanoparticles	Due to its metal ion dependence, easy hydrolysis, and low availability, the clinical application of disulfiram is limited. Accordingly, a novel metal organic framework (IRMOF3)-Zn^2+^ was developed, and disulfiram was incorporated in the IRMOF3. Folic acid was subsequently loaded on the surface, yielding IRMOF3 for targeted therapy of tumors.	nanotechnology	• folic acid • Zn(NO_3_)_2_·6H_2_O	The content of disulfiram in IRMOF3 was 25 mg/g	Intravenously applied for mice	Cancer	Cui et al. ^139^
11.	pH-responsive lipid-coated, polyethylene glycol (PEG) modified calcium phosphate nanoparticles co-loaded with Cu^2+^ and disulfiram	The limited water-solubility of disulfiram and systemic toxicity induced by exogenous Cu^2+^ hinder its practical application. To achieve improved synergetic tumor-responsive therapy with low side effects, pH-responsive lipid-coated calcium phosphate nanoparticles (LCP NPs) co-loaded with Cu^2+^ and disulfiram were constructed.	nanotechnology	• 1, 2-dioleoyl-*sn*-glycero-3-phosphate (sodium salt) (DOPA) • 1,2-dipalmitoyl-*sn*-glycero-3-phosphatidylcholine (DPPC) • 1,2-distearoyl-snglycero-3-phosphoethanolamine-*N*- (methoxy (polyethylene glycol)-5000) (DSPE-PEG_5k_) • cholesterol • CaCl_2_, • CuCl_2_·2H_2_O • Na_3_PO_4_·12H_2_O	The final loading capacity of disulfiram was determined to be ∼30 wt %	Intravenously applied for mice	Cancer	Li et al. ^140^
12.	Disulfiram (DIS)-loaded, Cu-doped zeolite imidazolate frameworks-8 (DIS-Cu/ZIF-8) nanoparticle followed by PEGylation (PEG-DIS-Cu/ZIF-8)	Off-target toxicity remains a major limitation of current cancer therapy, necessitating an alternative precision approach to treat cancers. Delivery of copper ions and disulfiram using a biodegradable metal organic framework nanoparticle demonstrates ultra-sensitive tumor microenvironment-responsive cargo release and *in situ* generation of various cytotoxic compounds, including Cu-chelate and reactive oxygen radicals.	nanotechnology	• zinc nitrate hexahydrate • cupric nitrate trihydrate • 2-Methylimidazole • PEG	DIS loaded (mg) on 10 mg Cu/ZIF-8: sample1: 1.796 sample2: 3.446 sample3: 10.163	N/A	Cancer	Zhang et al.^141^
13.	Smart nanosystem of disulfiram by coating with tannic acid (TA) and Cu^2+^ network, forming *in situ* bis (N,N-diethyldithiocanbamate)-copper complex	Co-delivery of disulfiram and Cu^2+^ to tumor tissues and generating a smart response to the tumor microenvironment (TME) are the focus of drug repurposing for the effective treatment of cancer. The anticancer efficacy of the resulting nano-prodrug can further be augmented by a continuous Fenton-like reaction.	nanotechnology	• tannic acid • CuCl_2_·2H_2_O	x	N/A	Cancer	Zhang et al.^142^
14.	Oral solid self-emulsifying drug delivery system (S-SEDDS) for concurrent delivery of drug combinations, including disulfiram	Non-oncology drug cocktail could overcome the shortage of anticancer therapeutics and help to reduce cancer-related mortality. The developed S-SEDDS presents an ideal system for concurrent oral delivery of non-oncology drug combinations.	nanotechnology	• Span 80 • soybean oil • Leciva S-95 • Poloxamer F108 (PF-108) • distilled water • Tween 80	15 mg disulfiram	oral	Cancer	Ardad et al.^143^
15.	Disulfiram-loaded water/oil emulsion	Disulfiram’s lipophilicity requires a different formulation for mice than for *in vitro* studies, in which it was dissolved in DMSO. To investigate disulfiram’s anticancer effects in an *in vivo* mouse model of high-risk neuroblastoma, a disulfiram-loaded emulsion was developed to deliver the highly liposoluble drug.	conventional pharmaceutical technology, w/o emulsion preparation	• Soybean oil • water • Span 80 • Tween 80	38 mg/mL	Intraperitoneally administered for mice	Cancer	Beaudry et al.^89^
16.	Molecular encapsulation of disulfiram with cyclodextrins	The clinical translation is limited by the disulfiram’s poor bioavailability. Therefore, the molecular encapsulation of disulfiram with cyclodextrins is evaluated to enhance the solubility and stability of the drug.	molecular encapsulation	• hydroxypropyl-β-cyclodextrin • randomly methylated-β-cyclodextrin • sulfobutylether-β-cyclodextrin	Stock solution with 1 mM disulfiram	N/A	Cancer	Benkő et al.^144^
17.	Low-intensity focused ultrasound (LIFU) and matrix metalloproteinase-2 (MMP-2) dual-responsive nanoplatform for the efficient utilization of the drug disulfiram	Disulfiram is unstable in acidic environments and rapidly degrades in the bloodstream, resulting in relatively low bioavailability. The developed nanoplatform is expected to promote the deep penetration and uniform dispersion of the bioavailable drug.	nanotechnology	• metalloproteinase-2 peptide • PEG_3000_-COOH • Poly (lactic-co-glycolic acid)-carboxylic acid (PLGA-COOH) • perfluoropentane	Drug loading content of nanoparticles is: 6.8%; and encapsulation efficiency is: 75.7%	Intravenously applied for mice	Cancer	Liu et al.^145^
18.	Microemulsion of disulfiram using cinnamon oil and Tween 80	The study aims to develop and optimize microemulsions (ME) through a quality-by-design (QbD) approach to improve the aqueous solubility and dissolution of poorly water-soluble drug disulfiram for repurposing in melanoma and breast cancer therapy	microtechnology	• Cinnamon oil • Tween 80	50 mg of disulfiram is used for preparation	N/A	Cancer	Mohapatra et al.^146^
19.	Oral solid self-emulsifying drug delivery system (S-SEDDS) for concurrent delivery of drug combinations, including disulfiram	Non-oncology drug cocktail could overcome the shortage of anticancer therapeutics and help to reduce cancer-related mortality. The developed S-SEDDS presents an ideal system for concurrent oral delivery of non-oncology drug combinations.	nanotechnology	• Span 80 • soybean oil • Leciva S-95 • Poloxamer F108 (PF-108) • distilled water • Tween 80	15 mg disulfiram	oral	Cancer	Ardad et al.^147^
20.	Disulfiram-loaded silk fibroin electrospun fibers, using hydroxypropyl-β-cyclodextrin inclusion complex of disulfiram	Disulfiram shows promising anticancer and antimicrobial activity, but its poor biopharmaceutical profile hinders its clinical use. This work aimed to develop DIS-loaded silk fibroin (SF) electrospun fibers for controlled release in the postsurgical resection cavity.	nanotechnology and molecular encapsulation	• silk fibroin • hydroxypropyl β-cyclodextrin • CuCl_2_	Disulfiram concentration was chosen to be 0.5 or 5.0% (*w*/*v*)	Implant	Cancer	Gonzalez-Prada et al.^148^
21.	Disulfiram dissolved in sterile 30% hydroxypropyl β-cyclodextrin	Increasing solubility of the drug	molecular encapsulation	• hydroxypropyl β-cyclodextrin	Stock solution: 50 mM	Intraperitoneally administered for mice	Infectious diseases	Potula et al.^101^
22.	Mucoadhesive drug delivery system for the local administration of disulfiram, composed of polyethylene glycol and carrageenan	Alternative formulations need to be developed to improve the efficacy of treatments administered via the vaginal route. Mucoadhesive disulfiram gels have the potential to be an effective alternative treatment for vaginal candidiasis	molecular encapsulation	• PEG-90M • carrageenan • hydroxypropyl-beta-cyclodextrin	0.5% or 1% *w*/*w*	Vaginal gel	Infectious diseases	Lajarin-Reinares et al.^112^
23.	Low-dose disulfiram rectal suppository containing cyclodextrins	Cyclodextrin encapsulation was investigated to improve the aqueous solubility of the hydrophobic drug. The presented drug delivery system relates to a novel preparation for rectal administration comprising a low-dose disulfiram with improved solubility and permeability by the PEG and hydroxypropyl-β-cyclodextrin (HPBCD) synergistic matrix.	molecular encapsulation	• hydroxypropyl-β-cyclodextrin • randomly methylated-β-cyclodextrin • PEGs • hard fat	30 mg	Rectal suppository	Infectious diseases	Benko et al.^149^
24.	Microenvironment-responsive Copper-based metal-organic frames nanoplatform for disulfiram activation	This platform releases disulfiram, binds to copper ions in the mildly acidic conditions of infected areas, converting disulfiram from nontoxic to toxic *in situ*, thereby inducing bacterial death and enhancing copper ion absorption.	nanotechnology	• copper acetate monohydrate • benzenetricarboxylic acid • dextran	30 mg disulfiram was added at preparation	N/A	Infectious diseases	Liu et al.^150^
25.	Disulfiram-loaded lactoferrin nanoparticles	Disulfiram-loaded lactoferrin nanoparticulate system was developed for combining the immunosuppressive activities of both disulfiram and lactoferrin.	nanotechnology	• lactoferrin	Drug loading content of nanoparticles is: 3.01 ± 0.04%; and encapsulation efficiency is: 56.86 ± 3.80%	Intravenously applied for mice	Inflammatory diseases	Ou et al.^151^
26.	Disulfiram loaded into poly(lactic-*co*-glycolic acid) (PLGA)-based nanoparticles and encapsulated in gelatin methacrylate microgels through a microfluidic device.	Disulfiram efficacy in treating osteoarthritis (OA) remains to be explored due to its poor water solubility and stability, which limit its use in OA treatment. A double-layer encapsulation approach is developed for intra-articular delivery of disulfiram for OA treatment in rat *in vivo* model.	nanotechnology	• PLGA • PLGA55k-*b*-PEG5k • Poly(vinyl alcohol • gelatin • methacrylic anhydride	0.1 mL 5 mg/mL DIS used in animal experiment	Intra-articular suspension	Inflammatory diseases	Liu et al.^152^
27.	Biocompatible liposomes incorporating a lung endothelial cell-targeted peptide (CGSPGWVRC) (LET) to produce disulfiram-loaded nanoparticles (DTP-LET@DIS NPs) for targeted delivery	Findings indicate that disulfiram is a promising treatment for inflammatory disorders. However, addressing the challenge posed by its intrinsic physicochemical properties, which hinder intravenous administration, and effective delivery to pulmonary vascular endothelial cells are crucial. Thus, targeted delivery by LET-functionalized nanoplatforms of repositioning disulfiram were developed.	nanotechnology	• dipalmitoylphosphatidylglycerol • dipalmitoylphosphatidylcholine • cholesterol • 1,2-distearoyl-*sn*-glycero-3-phosphoethanolamine • PEG2000 • lung endothelial cell-targeted peptide (CGSPGWVRC)lung endothelial cell-targeted peptide (CGSPGWVRC)	Encapsulation efficiency was 82.78%, and the drug loading content of nanoparticles was 24.87% when 2 mg of disulfiram was used at preparation	Intravenously applied for mice	Inflammatory diseases	Tian et al.^153^

ALG: alginate; CD: cyclodextrin; Cu(DDC)2: bis(diethyldithiocarbamate)-copper; DIS: disulfiram; DOPA: 1, 2-dioleoyl-*sn*-glycero-3-phosphate (sodium salt); DPPC: 1,2-dipalmitoyl-*sn*-glycero-3-phosphatidylcholine; DSPE-PEG5k: 1,2-distearoyl-snglycero-3-phosphoethanolamine-N- (methoxy (polyethylene glycol)-5000); HPBCD: hydroxypropyl-β-cyclodextrin; IRMOF3: folic acid-modified disulfiram/Zn-metal organic framework; LET: lung endothelial cell-targeted peptide; LIFU: low-intensity focused ultrasound; ME: microemulsions; MMP-2: matrix metalloproteinase-2; mPEG: monomethoxy (polyethylene glycol); mPLLA-PEG: methoxy b-poly(L-lactide) 2000- poly(ethylene glycol) 2000; NLC: nanostructured lipid carriers; NPs: nanoparticles; OA: osteoarthritis; PEG: polyethylene glycol; PEG_3000_-COOH: carboxylic acid-terminated polyethylene glycol; PLGA: Poly(lactic-co-glycolic acid); PLGA-COOH: Poly (lactic-co-glycolic acid)-carboxylic acid; PVP: polyvinylpyrrolidone; QbD: Quality-by-Design; RESS: rapid expansion of supercritical solutions; S-SEDDS: Oral solid self-emulsifying drug delivery system; SF: silk fibroin; SPIO: superparamagnetic iron oxide; TA: tannic acid; vitamin E-TPGS: d-α-tocopheryl polyethylene glycol 1000 succinate; W/O: water/oil emulsion; WPI: whey protein isolate; ZIF-8: zeolite imidazolate frameworks-8.

The conventional dosage forms of DIS are tablets, effervescent tablets, implants, and topical emulsions, whereas the new tendency in its repositioning is nanotechnology.^130^ However, the number of preclinical studies employing scalable drug formulation strategies to enhance DIS`s bioavailability and reduce systemic toxicity remains insufficient. Nanoformulations, predominantly developed for repurposing DIS in cancer treatment, but also in infectious and inflammatory diseases, enhance drug stability, protect against rapid *in vivo* degradation, and promote greater tumor accumulation^136^ (Table 2). Poly(lactic-co-glycolic acid) (PLGA) and metal-organic frameworks (MOFs) are preferred nanocarriers for DIS repurposing, while cyclodextrin (CD) molecular encapsulation is commonly used in both conventional and nanotechnology strategies. Yet none of these formulations has advanced beyond laboratory and preclinical stages of drug development. PLGA is an outstanding polymer, renowned for its mechanical strength, biocompatibility, and capacity to encapsulate a wide range of therapeutic agents, including small molecules, proteins, and nucleic acids.^154^^,^^155^ Several PLGA-based systems have already been approved by both the FDA and the European Medicines Agency (EMA) and are widely used in clinics for the treatment or diagnosis of diseases, no PLGA nanomedicine formulation is currently available on the global market.^154^^,^^155^

The drug-loading content of nanoformulation-based delivery systems is unsatisfactory (almost < 20%), requiring substantial amounts of nanomaterials. Excessive use of such nanomaterials, as pharmaceutical excipients, contravenes regulatory bodies' safety guidelines and risks severe side effects.^136^ Another drawback of drug development is the predominant emphasis on fundamental carrier exploration rather than comprehensive dosage form optimization (Table 2). Challenges arise when moving from benchtop to large-scale production because, unlike the conventional drug products, the efficacy and safety, as well as the unique drug delivery characteristics of each individual nanomedicine formulation, are a direct consequence of the physicochemical properties of the nanoparticles that carry the drug, which can be altered when adopting a larger scale production process.^154^ This yields non-scalable methodologies incompatible with current industrial pharmaceutical technologies and poses regulatory challenges. These limitations undermine the core advantages of drug repurposing strategies, including time- and cost-effectiveness, and hinder the extension of the life cycle for repositioning DIS.

## Discussion

### Shades of off-label use

DIS’s approved indication for alcohol dependence provides advantages, including a well-established tolerability profile, known pharmacokinetics, and low cost. However, these same characteristics reduce pharmaceutical industry incentives to fund expensive, large-scale clinical trials, creating a gap between preclinical findings and the evidence required for regulatory approval of new indications.^156^^,^^157^ Off-label use and clinical studies with DIS beyond alcohol use disorder frequently report tolerability issues. The growing scientific interest beyond its approved therapeutic application, the multiple potential of the drug, and the regulatory flexibility surrounding off-label prescribing bring both opportunities and challenges. Off-label prescribing without solid clinical validation may expose patients to uncertain benefit-risk profiles, particularly in complex areas such as oncology or infectious disease. The fragmented nature of international regulatory guidelines further complicates their integration into clinical practice.^158^ Case reports and preliminary studies, such as high-throughput screens and promising *in vitro* results, effectively highlight the potential efficacy of drug repurposing. Nonetheless, preclinical toxicity assessments via animal models and standardized clinical trials remain essential. These steps are bypassed due to intense social media-driven demand, which prioritizes speed over rigorous analysis; consequently, off-label DIS use proliferated for various diseases following its rapid promotion. Divergent international perspectives and regulations on off-label prescribing have rendered treatment protocols, applications, and consequences difficult to track; they typically emerge only when adverse events are reported or severe toxicities are published. The key benefit of off-label prescribing is addressing unmet medical needs where standard treatments fall short, thereby broadening medication access for vulnerable patient groups.^159^ This aligns with the European Committee’s report on off-label use, as studies confirm its prevalence in rare diseases, such as oncology and psychiatry, particularly among children and the elderly.^159^ Despite its growing clinical relevance, off-label prescribing raises important ethical, legal, and scientific concerns.^158^ In the European Union, for example, off-label use is not regulated centrally; each Member State has developed its own policy.^4^^,^^158^ In Hungary, prior authorization by the National Center for Public Health and Pharmacy (NNGYK) is required, based on a formal request and informed patient consent, thus providing greater regulatory oversight (Decree 44/2004).^4^ In Spain, the Royal Decree 1015/2009 establishes that the prescription of medicines off-label must be exceptional, limited to situations where there is no authorized alternative for the patient, and subject to his/her consent.^4^ By contrast, in Portugal and Romania, off-label prescription is allowed but not formally regulated, placing full responsibility on the prescribing physician. Regulatory strategies also differ in the United States; the FDA approves medicines only for specific indications, but physicians are allowed to prescribe them off-label when supported by scientific evidence. The main restriction is the prohibition of commercial promotion of off-label uses by pharmaceutical companies.^3^ In Japan, off-label prescribing is permitted at the discretion of the physician, though it is not automatically reimbursed, which may limit patient access. To address this, the Japanese Ministry of Health has created a mechanism that allows the approval of new indications based on international scientific literature, even in the absence of additional domestic clinical trials.^160^

### Recent status and future directions for disulfiram repositioning

**Figure 5:**
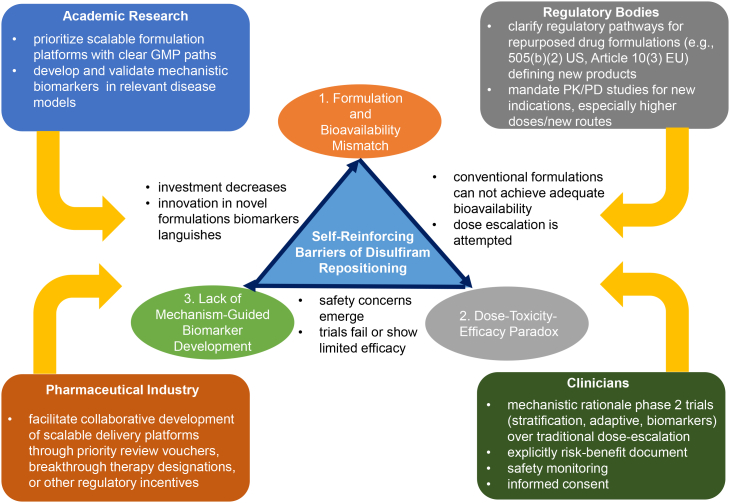
Self-reinforcing barriers and essential prerequisites for successful disulfiram repositioning in clinical practice This conceptual figure illustrates how three interconnected barriers—(1) formulation and bioavailability mismatch, (2) dose-toxicity-efficacy paradox, and (3) lack of mechanism-guided biomarker development—create a self-reinforcing cycle that hampers effective disulfiram repositioning. The diagram also outlines key, stakeholder-specific prerequisites for breaking this cycle, including the roles of academic researchers, the pharmaceutical industry, clinicians, and regulatory bodies in optimizing formulations, implementing mechanistic biomarker strategies, designing rational clinical trials, and clarifying regulatory pathways for repurposed products.

DIS represents an important case study in repurposing: extensive preclinical evidence and clinical use history are necessary but insufficient without addressing formulation, dosing, and biomarker barriers. While mechanistic insights, particularly in oncology, are gradually emerging, drug development efforts have not paralleled these advances. The integration of clinical trial data with preclinical mechanistic literature reveals three interdependent barriers to successful DIS repositioning (Figure 5). The transition from preclinical promise to clinical reality requires a coordinated strategy engaging academia, clinicians, the pharmaceutical industry, and regulatory bodies. While Table 2 documents extensive nanotechnology development, none have advanced beyond preclinical or early-stage laboratory testing. Current clinical trials continue to rely exclusively on oral conventional or effervescent tablets, despite clear evidence that DIS suffers from: poor aqueous solubility, rapid first-pass hepatic metabolism, low target tissue bioavailability, and insufficient copper complex formation at therapeutic doses when administered separately. The discrepancy between theoretical formulation advantages (demonstrated *in vitro*) and clinical reality (no approved formulations) reflects regulatory, manufacturing, and financial barriers to translating nanotechnologies into Good Manufacturing Practice (GMP) -compliant, reproducible, and cost-effective products suitable for clinical trials. Clinical trials employ doses even 4 times higher than approved alcoholism therapy, yet efficacy remains inconsistent. Safety signals at higher doses (hepatotoxicity, neurotoxicity) emerge without corresponding efficacy gains, suggesting that traditional linear dose escalation may be inappropriate for DIS. Adequate tissue concentrations may require formulation innovations rather than dose increases. Extensive preclinical mechanistic research identifies dozens of molecular targets and pathways. However, clinical trials have not incorporated mechanistic biomarkers to predict patient responsiveness (e.g., ALDH activity, copper metabolism capacity, and metallothionein status); enable adaptive trial designs with early stopping rules; allow post-hoc mechanistic analysis linking biomarkers to outcomes; and guide dose optimization based on individual pharmacokinetics. Without such biomarkers, clinical trials remain exploratory rather than hypothesis-driven, reducing power to detect genuine effects in heterogeneous populations. Success requires translating mechanistic insights into clinical tools, developing scalable products, and designing trials that test specific hypotheses in selected patient populations rather than broad exploratory studies. Until these prerequisites are met, off-label DIS use should be reserved for exceptional circumstances with thorough risk-benefit evaluation and informed consent emphasizing the limited clinical evidence base.

### Limitations of the study

Several limitations should be acknowledged, such as the search strategy, although systematic, may have undercaptured relevant studies due to semantic variability. The decision to focus on the term “off-label” may have excluded clinical trials or case series described under repositioning-related terminology. Records not mentioning the repositioning strategy may have been omitted due to the search keywords employed. Differences in study designs, populations, dosages, and endpoints across trials limited the possibility of direct comparison or meta-analysis. The heterogeneity observed in original articles also reflects the exploratory nature of most DIS repurpose efforts.

### Conclusions

This systematic review synthesizes evidence on DIS applications beyond alcohol dependence, integrating off-label and repurposed uses into a comprehensive preclinical-to-clinical data matrix. Off-label experiences provide key insights into dosing, safety, and toxicity, while repurposed studies offer mechanistic evidence and drug development perspectives. DIS holds strong potential for life cycle extension due to its multifaceted mechanisms and established clinical history, yet it has remained stagnant for over a decade without a repositioning paradigm shift. This review addresses a key literature gap: Although DIS repositioning features broad mechanistic appeal, limited systematic efforts have focused on real-world clinical use beyond alcoholism, which lacks double-blind controlled trials and scalable drug delivery systems. The findings highlight isolated but meaningful therapeutic benefits, tempered by tolerability issues and the absence of large-scale confirmatory trials. It also reinforces the need to distinguish experimental repositioning from true off-label use, particularly when evaluating clinical relevance and regulatory implications. Therapeutic enthusiasm must be balanced by rigorous safety assessments and realistic appraisals of clinical translatability. In-depth investments in pharmaceutical technology are essential for breakthroughs in alternative therapeutic areas. Future research should focus on well-designed, adequately powered clinical studies that incorporate pharmacological and pharmaceutical technology considerations to explore advanced and up-scalable delivery systems.

## Resource availability

### Lead contact

Requests for further information and resources should be directed to and will be fulfilled by the lead contact, Romána Zelkó (zelko.romana@semmelweis.hu).

### Materials availability

This study did not generate new unique reagents.

### Data and code availability

• All data reported in this paper will be shared by the lead contact upon request.
• This paper does not report original code.
• Any additional information required to reanalyze the data reported in this paper is available from the lead contact upon request.

## Acknowledgments

The authors received no financial support for the research, authorship, and/or publication of this article.

## Author contributions

Data curation, N.S.D.B.P.; conceptualization, R.Z.; formal analysis, B.M.B.; investigation, B.M.B.; methodology, B.M.B.; supervision, R.Z.; visualization, B.M.B. and I.S.; writing – original draft, B.M.B. and N.S.D.B.P.; writing – review and editing, R.Z. and I.S.

## Declaration of interests

The authors declare no competing interests.

## STAR★Methods

### Key resources table

**Table:** 

REAGENT or RESOURCE	SOURCE	IDENTIFIER
**Deposited data**		

Database for systematic review	PubMed, Scopus, Embase, Web of Science	The full search strategies are given in Supplemental Information. The included studies are presented in Table S1.
Clinical trial registers	ClinicalTrias.gov, WHO International Clinical Trials Registry Platform (ICTRP)	The full search strategies are given in Supplemental Information. The included studies are presented in Table 1.

**Software and algorithms**		

Microsoft 365 Excel	Microsoft Corporation	https://excel.cloud.microsoft/
Microsoft 365 PowerPoint	Microsoft Corporation	https://powerpoint.cloud.microsoft/
OriginPro 2018	OriginLab Corporation	https://www.originlab.com/
EndNote TM 20.6	Clarivate	https://endnote.com/
Adobe InDesign CC2014	Adobe.Inc.	https://www.adobe.com/apps/all/desktop/pdp/indesign

### Method details

#### Eligibility criteria

The review followed the PRISMA 2020 guidelines to ensure transparency and methodological rigor throughout all stages of the process.^161^ Original research articles reporting either preclinical (*in vitro* and *in vivo* results, demonstrating therapeutic intent in validated models of disease, with measurable outcomes indicative of pharmacological benefit) or clinical data (registered trials, observational studies and case reports), published within the last 10 years, in English were included. Studies were excluded if they were reviews, editorials, letters, conference materials or opinion pieces without original data; did not have DIS as the primary therapeutic agent; had no translational relevance or therapeutic context.

#### Information sources and search strategy

A comprehensive search of the literature was conducted between November and December 2025. Searches were performed across major scientific, bibliographic databases (PubMed, Scopus, Embase, Web of Science) using combinations of the terms “disulfiram” AND “off-label” OR “repurposing” OR “repositioning”. To ensure the identification of studies involving actual DIS administration in humans, the following clinical trial registries were also consulted: ClinicalTrials.gov and the WHO International Clinical Trials Registry Platform (ICTRP), using the drug name and the relevant disease-specific keywords. These restrictions ensured that the search captured studies with original and clinically or translationally relevant data, aligned with the scope of this review. The full search strategies for all databases and registers are presented in Supplementary Information.

#### Screening and data collection process

The research question was defined using the PICO framework: the population of interest included individuals with diseases other than alcohol dependence; the intervention was the off-label and repurposed use of DIS, either as monotherapy or in combination; comparators were not required due to the exploratory nature of most studies; and outcomes of interest included any evidence of therapeutic efficacy and/or safety. Each selected article was reviewed to extract data on indication, study design, population or model used, dosage, treatment duration, outcomes, adverse events, and whether co-treatment was used. The final synthesis was structured primarily by the clinical interest, derived by registered trials, and secondly by identified therapeutic areas and by type of evidence (clinical or preclinical) allowing for a critical appraisal of both translational potential and real-world application.

#### Risk of bias assessment

Due to heterogeneity of included studies (clinical trials, case reports, preclinical models), formal GRADE or ROB-2 scoring were not applied. Instead, critical appraisal was structured by clinical indication with emphasis on safety signals and clinical translatability.

### Quantification and statistical analysis

All literature identification was performed by EndNote20.6 (Clarivate, Philadelphia, USA). The raw data were collected using Excel App (Microsoft 365, Seattle, WA, USA). The data analyses were proceeded with OriginPro 2018 (OriginLab Corp., Microcal Software, Inc., Northampton, MA, USA) and figures were generated using PowerPoint (Microsoft 365, Seattle, WA, USA) or Adobe InDesign CC2014 (Adobe, San Jose, USA).

## Supplemental information

Document S1. The full search strategies for all databases and registers

Table S1. Summary of the studies included in the present systematic review (Excel)
