# Supplementary material for: Off-label and repurposed use of disulfiram beyond alcohol dependence: A systematic review of clinical and preclinical evidence
Source: iScience. 2026 Apr 3;29(5):115441. doi: 10.1016/j.isci.2026.115441 (PMC13122208; doi:10.1016/j.isci.2026.115441)
Supplement: Document S1. The full search strategies for all databases and registers [file mmc1.pdf]

iScience, Volume *29*

Supplemental information

Off-label and repurposed use of disulfiram beyond alcohol dependence: A systematic review of clinical and preclinical evidence

Beáta-Mária Benkő, Neuza Sofia De Brito Parreirinha, István Sebe, and Romána Zelkó

Supplementary Informations

The full search strategies for all databases and registers:

- PubMed:
  - query:(((disulfiram)AND((off-label) OR (repurposing) OR (repositioning)))
  - filters: in the last 10 years, Adaptive Clinical Trial, Case Reports, Classical Article, Clinical Study, Clinical Trial, Clinical Trial, Phase I, Clinical Trial, Phase II, Clinical Trial, Phase III, Clinical Trial, Phase IV, Clinical Trial Protocol, Controlled Clinical Trial, Legal Case, Meta-Analysis, Multicenter Study, Observational Study, Randomized Controlled Trial.
- Scopus
  - query: (TITLE-ABS-KEY (disulfiram) AND (TITLE-ABS-KEY (off-label) OR TITLE-ABS-KEY (repurposing) OR TITLE-ABS-KEY (repositioning)))
  - filters: AND PUBYEAR > 2014 AND (LIMIT-TO (DOCTYPE, "ar")) AND (LIMIT-TO (LANGUAGE, "English"))
- Embase:
  - query: 'disulfiram' AND ('off label' OR 'repurposing' OR 'repositioning')
  - filters: AND [2015-2025]/py AND ('article'/it OR 'clinical trial'/it)
- Web of Science:
  - query: ALL=(disulfiram) AND (ALL=(off-label) OR ALL=(repurposing) OR ALL=(repositioning))
  - filters: 2015-01-01 to 2025-11-25 (Publication Date) and Article (Document Type)
- ClinicalTrias.gov
  - query: intervention disulfiram
  - filter: last update posted from 01/01/2015 to 05/31/2025
- ICTRP
  - query: intervention disulfiram
  - filter: registration year > 2015
